# Associations between plasma sulfur amino acids and specific fat depots in two independent cohorts: CODAM and The Maastricht Study

**DOI:** 10.1007/s00394-022-03041-4

**Published:** 2022-11-02

**Authors:** Elena C. Tore, Amany K. Elshorbagy, Frans C. H. Bakers, Martijn C. G. J. Brouwers, Pieter C. Dagnelie, Simone J. P. M. Eussen, Jacobus F. A. Jansen, M. Eline Kooi, Yvo H. A. M. Kusters, Steven J. R. Meex, Thomas Olsen, Helga Refsum, Kjetil Retterstøl, Casper G. Schalkwijk, Coen D. A. Stehouwer, Kathrine J. Vinknes, Marleen M. J. van Greevenbroek

**Affiliations:** 1 Department of Internal Medicine, Maastricht University, Maastricht, The Netherlands; 2 CARIM School for Cardiovascular Disease, Maastricht University, Universiteitssingel 50, 6229 ER MD Maastricht, Postbus 616 6200, Maastricht, The Netherlands; 3 Department of Pharmacology, University of Oxford, Oxford, UK; 4 Department of Physiology, Faculty of Medicine, University of Alexandria, Alexandria, Egypt; 5 Department of Radiology and Nuclear Medicine, Maastricht University Medical Center, Maastricht, The Netherlands; 6 Department of Epidemiology, Maastricht University, Maastricht, The Netherlands; 7 CAPHRI Care and Public Health Research Institute, Maastricht University, Maastricht, The Netherlands; 8 MHENS School for Mental Health and Neuroscience, Maastricht University, Maastricht, The Netherlands; 9 Central Diagnostic Laboratory, Maastricht University Medical Center, Maastricht, The Netherlands; 10 Department of Nutrition, Institute of Basic Medical Sciences, University of Oslo, Oslo, Norway

**Keywords:** Sulfur amino acids, Adiposity, Regional fat distribution, Liver fat

## Abstract

**Purpose:**

Sulfur amino acids (SAAs) have been associated with obesity and obesity-related metabolic diseases. We investigated whether plasma SAAs (methionine, total cysteine (tCys), total homocysteine, cystathionine and total glutathione) are related to specific fat depots.

**Methods:**

We examined cross-sectional subsets from the CODAM cohort (*n* = 470, 61.3% men, median [IQR]: 67 [61, 71] years) and The Maastricht Study (DMS; *n* = 371, 53.4% men, 63 [55, 68] years), enriched with (pre)diabetic individuals. SAAs were measured in fasting EDTA plasma with LC–MS/MS. Outcomes comprised BMI, skinfolds, waist circumference (WC), dual-energy X-ray absorptiometry (DXA, DMS), body composition, abdominal subcutaneous and visceral adipose tissues (CODAM: ultrasound, DMS: MRI) and liver fat (estimated, in CODAM, or MRI-derived, in DMS, liver fat percentage and fatty liver disease). Associations were examined with linear or logistic regressions adjusted for relevant confounders with z-standardized primary exposures and outcomes.

**Results:**

Methionine was associated with all measures of liver fat, e.g*.,* fatty liver disease [CODAM: OR = 1.49 (95% CI 1.19, 1.88); DMS: OR = 1.51 (1.09, 2.14)], but not with other fat depots. tCys was associated with overall obesity, e.g., BMI [CODAM: *β* = 0.19 (0.09, 0.28); DMS: *β* = 0.24 (0.14, 0.34)]; peripheral adiposity, e.g., biceps and triceps skinfolds [CODAM: *β* = 0.15 (0.08, 0.23); DMS: *β* = 0.20 (0.12, 0.29)]; and central adiposity, e.g., WC [CODAM: *β* = 0.16 (0.08, 0.25); DMS: *β* = 0.17 (0.08, 0.27)]. Associations of tCys with VAT and liver fat were inconsistent. Other SAAs were not associated with body fat.

**Conclusion:**

Plasma concentrations of methionine and tCys showed distinct associations with different fat depots, with similar strengths in the two cohorts.

**Supplementary Information:**

The online version contains supplementary material available at 10.1007/s00394-022-03041-4.

## Background

Sulfur amino acids (SAAs) comprise the essential amino acid methionine and its derivatives [1, 2]. Methionine is converted into homocysteine in the transmethylation pathway. Subsequently, homocysteine can either be reconverted into methionine or undergo irreversible transsulfuration in the liver to form cystathionine and then cysteine, which may be used for the synthesis of proteins, taurine or glutathione. Homocysteine, cysteine and glutathione circulate bound to proteins or in their reduced or oxidized forms; the latter comprise dimers containing a disulfide bond between two homologous molecules or with other thiols. Total homocysteine (tHcy), total cysteine (tCys) and total glutathione (tGSH) refer to the sum of homocysteine, cysteine or glutathione fractions in plasma. For simplicity, we refer to all examined compounds within the SAA metabolic pathway, thus including cystathionine and the tripeptide glutathione, as ‘SAAs’.

Mounting evidence suggests that SAAs are related to obesity. Plasma tCys has been consistently and positively associated with body mass index (BMI) and whole-body fat mass [3–9] in human observational studies. For the other SAAs, some [4–7, 10–12], but not all [4–8, 13, 14], observational studies report positive correlations for methionine, tHcy and cystathionine with BMI or other measures of overall adiposity. Plasma levels of tGSH have generally shown inverse associations with BMI or fat mass [5, 15]. Moreover, observational studies have shown that tCys was associated with higher central fat mass, visceral adipose tissue (VAT) [7] and trunk fat-to-total fat ratio [5], and tGSH was associated with lower trunk fat-to-total fat ratio [5]. Positive correlations have also been observed between plasma methionine or tHcy and visceral or hepatic fat [16–21], although contrasting results have also been reported [14, 21–23].

The evidence from animal and in vitro studies supports these findings. Different body fat depots are known to confer different metabolic risk, with visceral and hepatic fat increasing the risk of insulin resistance and type 2 diabetes (T2D), cardiovascular disease (CVD) and some cancers [24, 25]. By contrast, a weaker cardiometabolic risk or even a beneficial effect has been suggested for subcutaneous adipose tissue (SAT) [26, 27]. Notably, methionine plays an important role in regulating hepatic fat metabolism, and impaired methionine metabolism in liver has been associated with fatty liver and related complications [28, 29]. Additionally, cysteine appears involved in preadipocyte differentiation and adipogenesis [9, 30]. Animal studies have further showed that dietary SAAs restriction decreases adiposity and liver fat accumulation [31].

Despite the consistent associations observed for particularly plasma tCys and obesity, comprehensive investigations to clarify the roles of plasma SAAs in regional body fat accumulation are lacking. Understanding the relationships between SAAs and specific fat depots can help identify novel preventive or therapeutic strategies for obesity and related metabolic diseases. Consequently, we examined the associations between plasma SAAs and various measures of overall obesity, peripheral and central adiposity and fatty liver in two Dutch populations.

## Methods

### Study populations

**Fig. 1:**
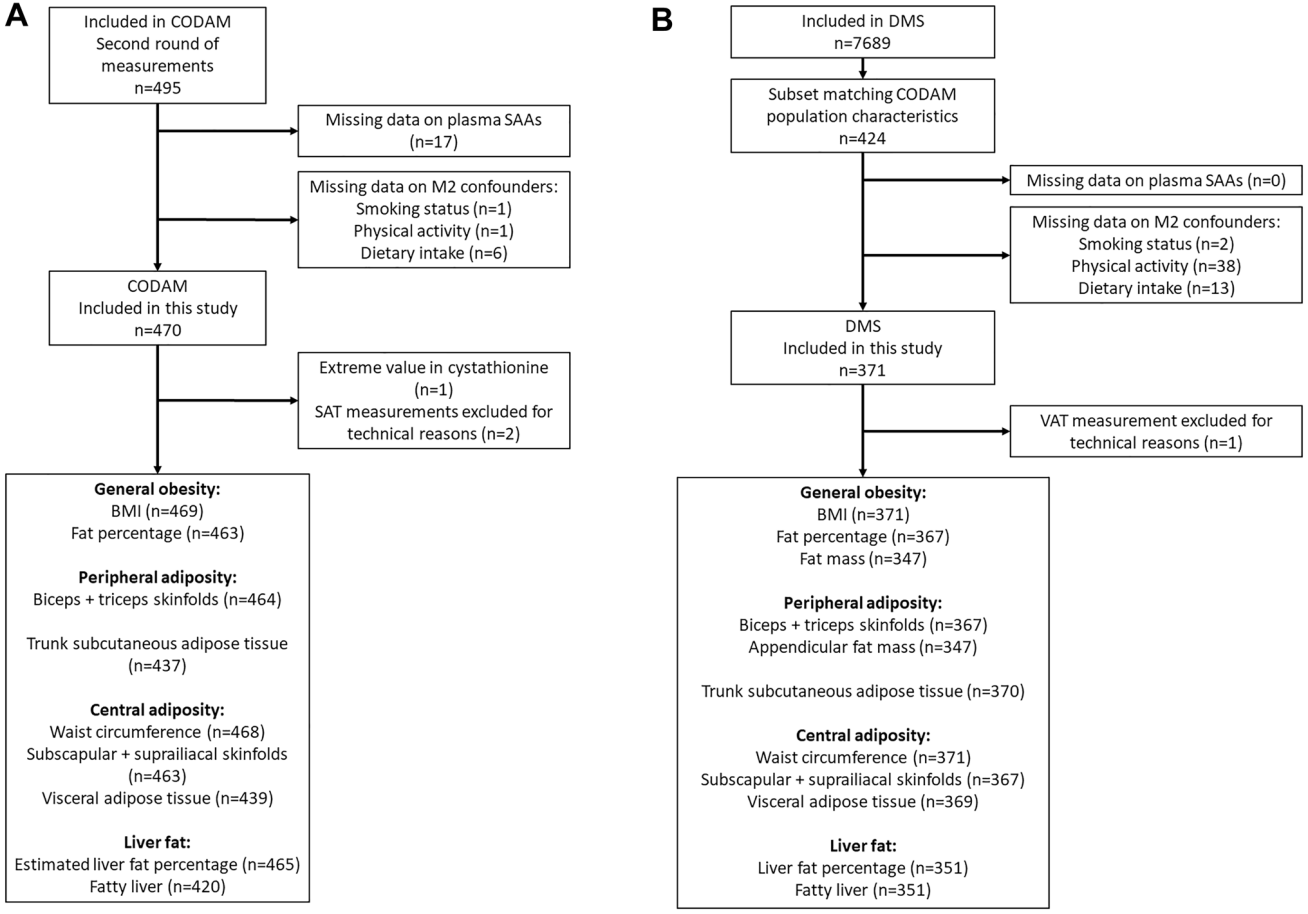
Flowchart of study participants. **A** CODAM, **B** DMS. *SAAs* sulfur amino acids, *SAT* subcutaneous adipose tissue, *VAT* visceral adipose tissue, *BMI* body mass index

The Cohort Study of Diabetes and Atherosclerosis Maastricht (CODAM) is a prospective cohort study established in the south of the Netherlands with the aim of examining the effects of glucose, lipids, lifestyle and genetics on cardiovascular complications [32]. Individuals were included if they were Caucasian, aged 40–70 years and met at least one of the following features: BMI > 25 kg/m^2^, positive family history for T2D, history of gestational diabetes, use of antihypertensive medication, postprandial blood glucose > 6.0 mM or glycosuria. This is a cross-sectional analysis of the 495 individuals who participated in the second round of measurements (July 2006–November 2009). Of these, 470 were included in the current study; individuals were excluded if data on plasma SAA concentrations (*n* = 17) or important covariates (*n* = 8) were missing (Fig. 1).

The Maastricht Study (DMS) is an observational prospective population-based cohort study. The rationale and methodology have been described previously [33]. In brief, the study focuses on the etiology, pathophysiology, complications and comorbidities of T2D and is characterized by an extensive phenotyping approach. Those eligible for participation were all individuals aged between 40 and 75 years and living in the South of the Netherlands. Participants were recruited through mass media campaigns and from the municipal registries and the regional Diabetes Patient Registry via mailings. Recruitment was stratified according to known T2D status, with an oversampling of individuals with T2D, for reasons of efficiency. For the present study, to obtain two comparable study populations, we selected a subset of 424 individuals that matched the distribution of age, sex and BMI of CODAM from the first 7689 participants who completed the baseline survey between November 2010 and December 2017. Of these, 371 were included in the current study; individuals were excluded if data on plasma SAA concentrations (*n* = 0) or other important covariates (*n* = 53) were missing (Fig. 1). The examinations of each participant were performed within a time window of 3 months.

Studies were approved by the local Medical Ethical Committee (CODAM: MEC05-170; DMS: NL31329.068.10). DMS was additionally approved by the Minister of Health, Welfare and Sports of the Netherlands (Permit 131,088-105,234-PG). All participants gave written informed consent.

### Plasma SAAs

Blood samples were collected after an overnight fast into pre-cooled EDTA tubes and kept on ice until centrifuged at 1950*g* for 15 min at 4 °C (within 3 h) to collect plasma. Plasma aliquots were stored at −80 °C until use. Plasma SAAs were assayed using liquid chromatography tandem-mass spectrometry (LC–MS/MS) with a modified version of a previously described method [34]. On the day of the SAA measurements, deuterium-labeled isotopes were added to EDTA plasma as internal standards, followed by dithioerythritol. Proteins were precipitated using perchloric acid, followed by centrifugation. The acid supernatant was diluted with an aqueous heptanesulfonic acid prior to LC–MS/MS, which was carried out using an LC-20ADXR Prominence LC system (Shimadzu, Kyoto, Japan) coupled to a QTRAP5500 mass spectrometer with a Turbo V ion source (Sciex, Framingham, MA, USA). Chromatographic separation was achieved on a Phenomenex Kinetex Core Shell C18 (100 × 4.6 mm, 2.6 μm) LC column (Torrance, CA, USA) with water and methanol gradient mobile phase spiked with formic acid [0.05%]. Positive mode multiple reaction monitoring was used for detection. Linear calibration curves of the peak area ratios of analytes and internal standards were used for quantification. In DMS, plasma concentrations of the branched-chain amino acids (i.e., leucine, isoleucine and valine, BCAAs) and of tyrosine were determined concurrently.

### Assessment of obesity and fat depots

Anthropometric characteristics were measured by trained staff. To calculate BMI (kg/m^2^), height (cm) was measured with a wall-mounted stadiometer, while body weight (kg, rounded to the nearest 0.1 kg) was assessed with subjects standing on calibrated electronic scales and wearing light clothes. Waist circumference (WC, cm) was measured with a flexible tape laid midway between the lateral lower rib margin and the anterior superior iliac spine. Thickness (mm) of the bicipital, tricipital, subscapular and suprailiac skinfolds was measured with a skinfold caliper (Servier, Neuilly–sur-Seine, France) and used to calculate whole-body fat percentage [35] and peripheral (sum of bicipital and tricipital skinfolds, SKF_BI-TRI_) and central (sum of subscapular and suprailiac skinfolds, SKF_SS-SI_) adiposity.

In CODAM, abdominal adipose tissue thickness (SAT_US_ and VAT_US_, mm) was calculated as the mean of three ultrasound measurements (US, ALT-Ultramark 4 plus ultrasound system, Bothell, WA, USA) taken in the middle and the two sides of the abdominal wall at the end-expiratory stage of normal respiration at the level of WC assessment [36, 37]. SAT_US_ was defined as the distance between the skin and the abdominal muscles (i.e., linea alba) and VAT_US_ as the distance between the peritoneum and the anterior of the vertebrate body [38].

In DMS, abdominal adipose tissue areas (SAT_MRI_ and VAT_MRI_, cm^2^) were assessed at the top level of the fourth lumbar vertebral body with a 3.0 Tesla magnetic resonance imaging (MRI) scanner (MAGNETOM Prismafit, Siemens Healthineers, Erlangen, Germany). Participants were positioned supine and a single-slice T_1_-weighted turbo spin echo MRI image in transverse plane was acquired during a breath-hold with body matrix and spine radiofrequency coils. Single-slice MR images at the level of the fourth lumbar vertebra are accurate for representing the total amount of SAT and VAT (Pearson’s *r* ≥ 0.89) [39]. The acquisition parameters were: echo time = 35–38 ms, repetition time = 550 ms, field-of-view = 384–500 × 312–406 mm^2^, slice thickness = 8 mm, acquired voxel size = 2.0 × 2.0 × 8.0 mm, and GRAPPA with a reduction factor of 2. SAT_MRI_ and VAT_MRI_ were quantified by trained staff using dedicated semi-automatic software (Quantib Abdominal Segmentation Tool [QAST], Quantib, Rotterdam, the Netherlands) based on a Gaussian curve fit to distinguish between fatty and non-fatty tissue with manual review of each segmentation analysis. The algorithm used by QAST is identical to the validated Hippofat software [40–42], but it allows for more efficient data handling and analysis of liver fat within one software environment. Moreover, in DMS, whole-body dual-energy X-ray absorptiometry (DXA, Discovery DXA scanner, Hologic, Zaventem, Belgium) was used to obtain the amounts (g) of fat mass in the whole body, arms, legs and trunk, in accordance with the provider’s instructions [43]. For the current study, the amount of fat in the arms and legs was summed up to obtain the total limb fat mass (Limb_FM_). Moreover, DXA-derived total-body lean mass (g, excluding bone mass) was assessed. For logistic reasons, MRI and DXA assessments and collection of plasma and other characteristics were not always concurrent. Therefore, the lag time between these assessments was noted.

In CODAM, liver fat percentage (eLF%) was estimated using the equation developed by Kotronen et al. [44]. This equation is based on the levels of liver enzymes, fasting insulin levels and presence of metabolic syndrome and T2D, and was validated against magnetic resonance spectroscopy (MRS) [44]. Furthermore, liver images were acquired with a US system (ALT-Ultramark 4 plus ultrasound system, Bothell, WA, USA) with a C7-4 and C4-2 transducer, as previously described [36]. Standardized images of the liver and right kidney were recorded on videotape and examined by an independent radiologist unaware of the individual's clinical characteristics. Diagnosis of fatty liver was established by conventional criteria, i.e., increased echogenicity (“bright liver”), posterior beam attenuation and decreased visualization of hepatic blood vessels [45, 46]. For the current study, fatty liver images were used to discriminate between no-to-mild and moderate-to-severe fatty liver.

In DMS, liver fat percentage (LF%) was measured through Dixon MRI using a 3.0 Tesla MRI system (MAGNETOM Prismafit, Siemens Healthineers, Erlangen, Germany) with body matrix and spine radiofrequency coils, as previously described [47]. Briefly, transversal two-dimensional T_2_-weighted true fast imaging with steady-state free precession (T_2_w TRUFI) images and, afterward, during a breath-hold, transversal two-dimensional turbo spin echo Dixon MR images were acquired through the liver. Three regions of interest positioned to avoid any artifact or visible structure in the liver were drawn by trained staff on the T_2_w TRUFI images. Subsequently, these regions of interest were copied to the water and fat Dixon MR images to calculate the intrahepatic lipid fraction. LF% was expressed as the ratio CH_2_/H_2_O (*100%). This method was calibrated and validated against proton MRS, the gold standard to non-invasively quantify liver fat content, in 36 participants. After calibration, the intra-class correlation coefficient was 0.99 (95% confidence interval (95% CI) 0.98, 0.99). In addition, we computed fatty liver as a dichotomous variable to discriminate between participants with and without clinically relevant fatty liver disease. The cutoff value was set at 5.89%, based on the definition of non-alcoholic fatty liver disease as intrahepatic lipid content of ≥ 5.56% and adapted for liver fat content expressed as CH_2_/H_2_O (0.0556/(1−0.0556) = 0.0589) [47, 48].

### Covariate assessment

Dietary intake during the preceding 12 months was collected in CODAM [49] and DMS [50] with self-reported validated food frequency questionnaires. Smoking behavior was self-reported and categorized as never, former or current smoker. Habitual physical activity (total of all activities combined per day) was assessed in CODAM with the Short Questionnaire to Assess Health-Enhancing Physical Activity (SQUASH; [51]) and in DMS with the Community Healthy Activities Model Program for Seniors Physical Activity Questionnaire for Older Adults (CHAMPS; [52]).

Glucose concentration was measured in fasting plasma after a standard 75 g oral glucose tolerance test with venous blood sampling [33]. Glucose metabolism status was defined according to the World Health Organization (WHO 1999 [53] in CODAM; WHO 2006 [54] in DMS) as normal glucose tolerance, prediabetes (impaired fasting glucose and impaired glucose tolerance combined), T2D and other type of diabetes. Total cholesterol, HDL cholesterol and triglycerides were determined in EDTA plasma (CODAM) or serum (DMS) using standard clinical chemistry methods. Serum creatinine levels were determined in CODAM with a Jaffé diagnostic test or with an enzymatic method (Roche Diagnostics, Mannheim, Germany), with values re-calibrated as previously explained [32]; in DMS, a Jaffé method was used (with two instruments due to a change of supplier, the Beckman Synchron LX20, Beckman Coulter Inc, and the Roche Cobas 6000, F. Hoffmann-La Roche Ltd).

Use of antihypertensive drugs or glucose-lowering or lipid-modifying medication (yes or no) was self-reported. Prior CVD was defined, in CODAM, as the presence of signs of myocardial infarction (i.e., Minnesota codes 1–1 or 1–2) or ischemia (i.e., Minnesota codes 1–3, 4–1, 4–2, 4–3, 5–1, 5–2, 5–3 or 7–1) on a 12-lead electrocardiography. In DMS, prior CVD was defined as a self-reported history of myocardial or cerebrovascular infarction, hemorrhage, percutaneous artery angioplasty of or vascular surgery on the coronary, abdominal, peripheral or carotid arteries. In CODAM, insulin resistance was quantified using the updated Homeostasis Model Assessment (HOMA2-IR) calculator (www.dtu.ox.ac.uk); no data on insulin resistance was available for this DMS subset.

Glomerular filtration rate (eGFR) was estimated using the short Modification of Diet in Renal Disease equation [55]. The hemolytic samples were identified based on visual inspection.

### Statistical analysis

Mean and standard deviation (SD) or median and interquartile range (IQR) were used to describe normally distributed or skewed continuous variables, respectively, while number and percentage were used for categorical variables. In CODAM, we identified one outlier in plasma cystathionine (8.1 µmol/l), where the median [IQR] of the rest of the population was 0.23 [0.17, 0.32]. This improbable value was excluded from all analyses.

Associations were investigated in linear or logistic regressions with z-standardized continuous primary exposures (i.e., SAAs) and outcomes (i.e., overall obesity: BMI, fat percentage, fat mass; subcutaneous peripheral adiposity: SKF_BI-TRI_ and Limb_FM_; subcutaneous abdominal adiposity: SAT; central adiposity: WC, SKF_SS-SI_, VAT; liver fat: eLF%, LF%, fatty liver). The linearity of the associations was examined with generalized additive models, which use penalized-regression splines to model the data; no significant departure from linearity was detected. Covariate selection was based on a literature-informed directed acyclic graph [56] (supplemental Fig. S1). Associations were first adjusted for age (years), sex (men/women) and glucose metabolism status (normal/prediabetes/T2D) (model 1). In model 2, additional adjustment was made for smoking status (never/former/current), alcohol (g/d) and coffee consumption (g/d), physical activity (arbitrary units in CODAM and hour/week in DMS) and the progenitor SAA (i.e., the SAA immediately preceding the primary exposure in the causal path, in SD); since methionine is an essential amino acid, no other SAA was included in analyses with methionine as primary exposure. In models with methionine or tCys as main exposure, model 2 was additionally adjusted for protein intake (percentage of energy from protein, E%) and total energy consumption (kcal/d). When MRI or DXA-derived fat measures were the outcome, model 2 was additionally adjusted for the lag time (days) between assessments. Additionally, height (m) was added to analyses with DXA-derived fat measures as outcomes. Finally, in model 3, we additionally adjusted for SAAs that were not yet in model 2, for protein intake and total energy intake, if not yet in model 2, and additionally for BMI in models with measurements of liver fat as outcome. Model 2 was considered the main model because it has the lowest combined risk of over-adjustment and residual confounding.

Several sensitivity analyses were performed to assess the robustness of our results, all based on model 2. We repeated the analyses replacing protein intake (E%) and total energy consumption by total protein intake (g, model 2a). Since hemolysis may affect the measurements of plasma concentrations of some SAAs, especially tGSH [57], we excluded participants with samples categorized as hemolytic (model 2b). We additionally adjusted for proxies of muscle mass, a source of plasma amino acids in the fasted state [58]: serum creatinine (model 2c) and lean mass in DMS (model 2d). We also repeated the analyses of model 2 in a DMS sample without missing data on lean mass, to verify whether variations in the estimates obtained with and without adjustment for lean mass were due to the smaller sample size (model 2e). We additionally adjusted for prevalent health conditions, including CVD, plasma triglycerides and total-to-HDL cholesterol ratio, systolic blood pressure, use of lipid-modifying, glucose-lowering or antihypertensive medication and kidney function (model 2f), or for insulin resistance in CODAM (model 2g). The analyses in models 2c, 2d, 2f and 2g were potentially over-adjusted since it cannot be excluded that creatinine levels, lean mass or prevalent health conditions were affected, at least partly, by overall obesity and unhealthy fat accumulation [59–62]. Moreover, we additionally adjusted for plasma concentrations of BCAAs and/or tyrosine, which are correlated with plasma SAA concentrations and have been associated with obesity and fatty liver (e.g., [63, 64]*,*) (models 2 h–j). These analyses might be over-adjusted, since no known direct metabolic pathway links these amino acids and thus their correlation might be due to other factors. Finally, we explored the interaction of methionine, tHcy or tCys with sex on each outcome (model 2g). These three SAAs were selected based on reported sex differences in hepatic one-carbon metabolism [65]. In case a statistically significant interaction was found in either cohort, sex-stratified analyses are presented for both cohorts. Additionally, based on a previously noted significant interaction between tCys and sex in relation to BMI (stronger association in women than men) [4], we present sex-stratified analyses of the associations between tCys and BMI regardless of statistical significance in the present cohorts.

All analyses were performed using R (version 4.0.5). Statistical significance was set at *p* < 0.05.

## Results

### Characteristics of the study populations

Participants’ characteristics were largely comparable between cohorts (Table 1). There were fewer women than men (38.7% and 46.6% women in CODAM and DMS, respectively), and a comparable distribution of participants with normal glucose tolerance, prediabetes and T2D. The age distribution was similar between cohorts, although there was a slightly higher age range in CODAM (67 years [61, 71]) than in DMS (63 years [55, 68]).

**Table 1:** Characteristics of study participants by cohort

	CODAM (*n* = 470)		DMS (*n* = 371)	
	Assessment, unit	Mean (SD), median [IQR] or *n* (%)	Assessment, unit	Mean (SD), median [IQR] or *n* (%)
Sex	% men	288 (61.3%)	% men	198 (53.4%)
Age	Years	67 [61, 71]	Years	63 [55, 68]
Glucose metabolism status	%		%	
Normal glucose tolerance		192 (40.9%)		207 (55.8%)
Prediabetes		115 (24.5%)		62 (16.7%)
Diabetes mellitus		163 (34.7%)		102 (27.5%)
Smoking status	%		%	
Never		117 (24.9%)		135 (36.4%)
Former		282 (60%)		202 (54.4%)
Current		71 (15.1%)		34 (9.2%)
Total calorie intake	kcal/day	2030 [1697, 2461]	kcal/day	2170 (618)
Total protein intake	g/d	77.9 [64.9, 93.3]	g/d	86.3 (24.2)
Methionine	Plasma, µmol/l	22 [19.9, 24.3]	Plasma, µmol/l	23.4 [21, 25.7]
Total homocysteine	Plasma, µmol/l	10.3 [8.2, 12.7]	Plasma, µmol/l	10.8 [9.1, 13.3]
Cystathionine	Plasma, µmol/l	0.23 [0.17, 0.32]	Plasma, µmol/l	0.2 [0.14, 0.28]
Total cysteine	Plasma, µmol/l	284 [261.4, 306.4]	Plasma, µmol/l	314.6 [289.4, 339.3]
Total glutathione	Plasma, µmol/l	4.84 [4.1, 6.3]	Plasma, µmol/l	3.66 [3.03, 4.93]
Body mass index	kg/m^2^	28.1 [25.8, 30.7]	kg/m^2^	28 [25.6, 30.5]
Body fat percentage	%	33.9 (6.6)	%	35.7 (7.7)
Whole-body fat mass			DXA, kg	29.6 (8.4)
Biceps and triceps skinfolds	mm	25.3 [18.3, 38.1]	mm	29 [20, 43.3]
Limb fat mass			DXA, kg	12.3 [9.8, 15.1]
Subcutaneous abdominal fat	US, mm	20.3 [14.1, 27.4]	MRI, cm^2^	221 [168, 292]
Waist circumference	cm	101 (12.1)	cm	99 (12.7)
Suprailiac and subscapular skinfolds	mm	43.8 [35.9, 53.1]	mm	47 [37, 66]
Visceral fat	US, mm	80.7 [61.7, 96.4]	MRI, cm^2^	191 [126, 269]
Liver fat	eLF, %	4.8 [2.55, 7.89]	MRI, %	4.17 [2.59, 7.42]
Fatty liver disease	US, % yes	221 (47.0%)	MRI, % yes	115 (32.7%)
Creatinine	Serum, µmol/l	102.1 (10.09)	Serum, µmol/l	83.06 (17.52)
Lean mass			DXA, kg	52.1 [44.2, 60.7]
Physical activity	SQUASH, arbitrary score	6150 [3775, 9142.5]	CHAMPS, h/week	12.75 [8.5, 18.75]

*DXA* dual-energy X-ray absorptiometry, *US* ultrasound, *MRI* magnetic resonance imaging, *eLF* estimated liver fat, *SQUASH* Short Questionnaire to Assess Health-Enhancing Physical Activity, *CHAMPS* Community Healthy Activities Model Program for Seniors Physical Activity Questionnaire for Older Adults

### Crude associations

Crude associations among all studied exposures and outcomes are reported in supplemental Fig. S2.

#### Crude associations among SAAs

There were positive associations between methionine and cystathionine, and among tHcy, cystathionine and tCys, while tGSH was inversely associated with tCys. Effect sizes were generally small to medium.

#### Crude associations among adiposity measures

There were positive associations between measures of overall obesity and measures of fat depots, except for whole-body fat percentage and VAT (*β* = 0.02 in both cohorts). Relatively strong associations were also found among specific fat depots. Weaker associations were found between WC or VAT and measures of peripheral adiposity. VAT and SAT were inversely associated in CODAM (*β* = − 0.21), but positively associated in DMS (*β* = 0.18).

#### Crude associations between SAAs and adiposity measures

Plasma SAAs were generally positively associated with adiposity measures, with small-to-medium effect sizes (*β* = 0.1–0.21 in CODAM, and *β* = 0.1–0.29 in DMS). Inverse or null associations were found for methionine and all measures of whole-body fat, peripheral fat depots and SAT, and for tHcy and fat percentage, SKF_BI-TRI_ and SAT. tGSH showed either null or inverse associations with all adiposity measures.

### Associations of plasma SAAs with adiposity measures

**Fig. 2:**
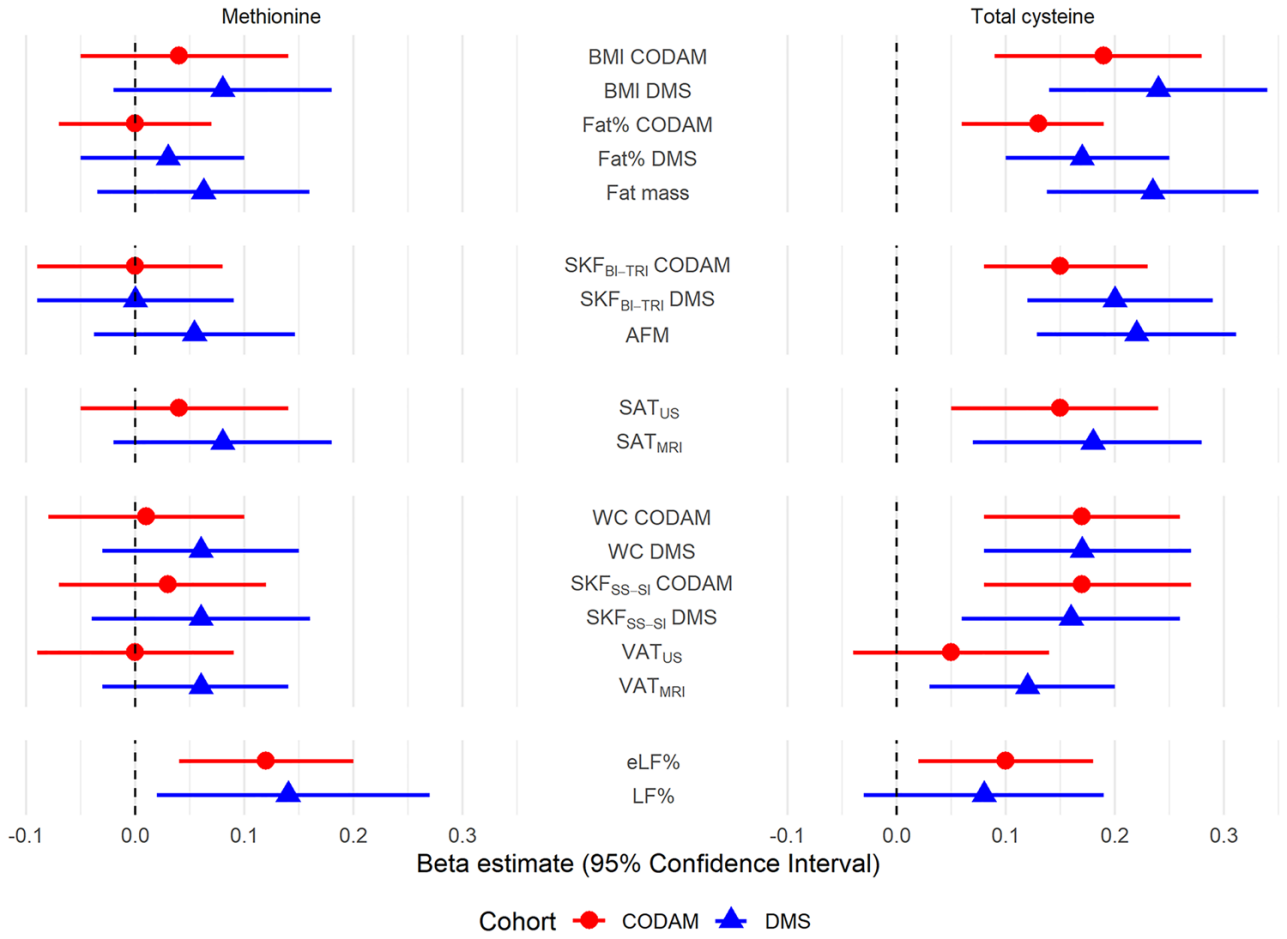
Associations of plasma methionine and tCys with different fat depots. In CODAM, *n* = 437–469, in DMS *n* = 347–371. Adjusted for age, sex, glucose metabolism status, smoking status, alcohol and coffee consumption, physical activity, plasma cystathionine (in models with tCys as main exposure), protein intake and total energy consumption, height (in models with DXA-derived measures as outcome) and, were applicable, for the lag time before MRI and DXA scans. *BMI* body mass index, *SKF*_*BI-TRI*_ sum of bicipital and tricipital skinfolds, *Limb*_*FM*_ limb fat mass, *SAT*_*US*_ subcutaneous adipose tissue by ultrasound, *SAT*_*MRI*_ subcutaneous adipose tissue by magnetic resonance imaging, *WC* waist circumference, *SKF*_*SS-SI*_ sum of subscapular and suprailiac skinfolds, *VAT*_*US*_: visceral adipose tissue by ultrasound, *VAT*_*MRI*_ visceral adipose tissue by magnetic resonance imaging, *eLF%* estimated liver fat percentage, *LF%* liver fat percentage

Estimates and 95% CIs of the adjusted associations between methionine and tCys and measures of overall obesity, peripheral and central adiposity, and liver fat, are presented in Fig. 2. Detailed information on the associations between all plasma SAAs and all adiposity-related outcomes is provided in supplemental Table S1. Results of the sensitivity analyses are shown in supplemental Tables S2–S5. Unless otherwise specified, estimates from model 2 are reported below.

### Methionine

Plasma methionine was not associated with any measure of overall obesity, peripheral or central adiposity. By contrast, a positive association was found with eLF% in CODAM [*β* = 0.12 (0.04, 0.20)], which was only modestly attenuated after adjusting for all other SAAs and BMI [*β* = 0.09 (0.01, 0.16)]. Moreover, participants with higher methionine levels had higher odds of fatty liver [OR = 1.49 (1.19, 1.88)]. Likewise, in DMS, there were positive associations with LF% [*β* = 0.14 (0.02, 0.27)] and fatty liver [OR = 1.51 (1.09, 2.14)].

In sensitivity analyses, after additional adjustment for BCAAs and/or tyrosine in DMS, the associations between methionine and liver fat were attenuated (Table S5). Other sensitivity analyses had little effect on the results (Tables S2–S6).

### Total cysteine

We found positive associations between tCys and BMI [CODAM: *β* = 0.19 (0.09, 0.28); DMS: *β* = 0.24 (0.14, 0.34)], fat percentage [CODAM: *β* = 0.13 (0.06, 0.19); DMS: *β* = 0.17 (0.10, 0.25)] and total fat mass in DMS [*β* = 0.23 (0.14, 0.33)]. Positive associations were also found between tCys and SKF_BI-TRI_ [CODAM: *β* = 0.15 (0.08, 0.23); DMS: *β* = 0.20 (0.12, 0.29)], Limb_FM_ in DMS [*β* = 0.22 (0.13, 0.31)] and SAT [CODAM: *β* = 0.14 (0.05, 0.23); DMS: *β* = 0.18 (0.07, 0.28)]. Moreover, tCys was positively associated with WC [CODAM: *β* = 0.16 (0.08, 0.25); DMS: *β* = 0.17 (0.08, 0.27)], SKF_SS-SI_ [CODAM: *β* = 0.17 (0.08, 0.27); DMS: *β* = 0.16 (0.06, 0.26)] and with VAT in DMS but not in CODAM [CODAM: *β* = 0.05 (− 0.04, 0.14); DMS: *β* = 0.12 (0.03, 0.20)].

There was a positive association between tCys and eLF% in CODAM [*β* = 0.10 (0.02, 0.18)], which was attenuated and non-significant after controlling for all other SAAs and BMI [*β* = 0.02 (− 0.05, 0.10)]. Similarly, no associations were found with fatty liver in CODAM [OR = 1.14 (0.92, 1.43)] or with LF% in DMS [*β* = 0.08 (− 0.03, 0.19)]. In DMS, no association of tCys with fatty liver was found after adjusting for all other SAAs and BMI [OR = 1.04 (0.76, 1.42)].

In sensitivity analyses, after additional adjustment for lean mass in DMS, the associations between tCys and adiposity measures were attenuated (Table S4, model 2d). Other sensitivity analyses, including additional adjustment for serum creatinine, had little effect on the results (Tables S2–S6).

### Other SAAs

Associations of the other three SAAs with adiposity measures were less strong and inconsistent between cohorts. Plasma tHcy was not associated with any outcome in CODAM, but was positively associated with overall obesity, peripheral subcutaneous adiposity, WC and VAT in DMS. However, no association remained after additional adjustments for other SAAs (Table S1).

Cystathionine was positively associated with BMI, WC and liver fat in both CODAM and DMS but estimates were substantially reduced after adjusting for all other SAAs (Table S1).

Plasma tGSH was positively associated with SAT and inversely associated with VAT in CODAM, while no association was found in DMS. After excluding hemolytic samples, we found inverse associations with BMI and WC in CODAM and non-significant trends in DMS (Table S3).

## Discussion

In this comprehensive examination of the relations between plasma SAAs and adiposity in two Dutch populations enriched with individuals with prediabetes and T2D, plasma methionine was not associated with measures of overall, peripheral or central adiposity, but it was positively associated with measures of liver fat. By contrast, plasma tCys was associated with overall adiposity, abdominal SAT, and measures of peripheral and central adiposity, while the associations with VAT and liver fat were less consistent between cohorts. Finally, no clear associations of plasma tHcy, cystathionine or tGSH and adiposity were identified.

A role for SAAs in energy metabolism has long been hypothesized. Mice on a methionine-restricted diet, which results in lower serum methionine and tCys, have reduced total-body and visceral fat [66]. The observation that these effects were largely reversed by cysteine supplementation [67, 68], and that administration of cysteine in physiologic concentrations drives preadipocyte differentiation and adipogenesis, stimulating lipid accumulation and lipid droplet size in vitro [9, 30], suggests that high circulating tCys concentrations might promote adiposity. Human observations support this hypothesis. Plasma tCys has been consistently associated with BMI and body fat mass [3–9]. Only a few studies examined its relationship with central adiposity, reporting positive correlations with WC [4], trunk fat-to-total fat ratio [5], and central fat mass [7], but inconsistent associations with VAT [7, 14]. In the present study, we extend these observations by additionally showing positive associations with peripheral adiposity.

The associations of tCys with VAT and measures of liver fat were inconsistent between cohorts, and weaker compared to the associations with SAT and measures of peripheral adiposity, suggesting that tCys may be primarily related to expansion of abdominal and peripheral SAT. The distinct gene-expression profiles of human SAT and VAT may be one factor underlying their different susceptibility to circulating tCys. For example, it has been shown that the activity of the nuclear receptor peroxisome proliferator activated receptor gamma (PPARG) is more prominent in human SAT than VAT [69]. Additionally, PPARG expression, with consequent lipid accumulation, increased after cysteine supplementation in vitro and in vivo [9, 30, 68]. Further investigations are nonetheless required to confirm the different effect of tCys on SAT and VAT as well as the mechanism of action.

Additional adjustment for lean mass substantially attenuated the associations of tCys with all adiposity measures. Although not consistently adjusted for in previous studies [3–6, 14], lean mass could be considered a confounder of these associations because, as a proxy for skeletal muscle mass, it represents a source of plasma amino acids during the fasted state, when amino acids are released in the circulation to support protein synthesis and gluconeogenesis in other tissues [58]. In addition, a higher lean mass increases the resting energy expenditure potentially reducing fat accumulation [58, 61]. However, lean mass is also affected by greater fat mass in two, opposite, ways. On one hand, the skeletal muscle mass increases in people with obesity to support a greater body weight [61], while on the other, fat mass and especially VAT promote muscle atrophy [59–61]. In this study, considering that median BMI values of the two populations are in the overweight range, it can be argued that the effect of adiposity on lean mass is predominant. Consequently, its inclusion in sensitivity analyses may have resulted in over-adjustment [70].

In contrast to tCys, plasma methionine was not associated with any measure of overall obesity, peripheral or central adiposity, but it was positively associated with measures of liver fat. Methionine is the precursor of S-adenosylmethionine (SAM), the key methyl donor for the synthesis of phosphatidylcholine, which is required for the export of very low-density lipoproteins from the liver [71]. Downregulation of MAT1A [18], with consequent reduced synthesis of SAM from methionine, and lower rates of methionine transmethylation [72] are often present in non-alcoholic fatty liver disease patients. Our findings of a positive, linear association between methionine and measures of liver fat are consistent with observations that methionine concentrations were higher in patients with fatty liver disease compared to healthy controls [19, 20]. Additionally, our results suggest that this association is independent of several confounding factors. Overall, the available evidence suggests that an impaired methionine metabolism, with consequent excessive or deficient plasma methionine concentrations, results in intrahepatic lipid accumulation [28, 29]. By contrast, SAA-restricted diets, which are achieved by lowering the intake of methionine and/or cysteine to levels close to minimum requirements, have been proven beneficial in reducing liver fat accumulation in rodents [31, 73]. Furthermore, methionine restriction in humans resulted in lower liver fat in people with obesity and metabolic syndrome [74]. The lack of significant associations between methionine and liver fat we saw after additional adjustment for plasma concentrations of BCAAs and/or tyrosine is therefore in contrast with the overall evidence. Future studies should clarify whether these results might be due to specific biological reasons, such as, e.g., a common amino acid transporter [75], or to statistical over-adjustment. Of note, we detected mild-to-moderate multicollinearity in these analyses, suggesting that over-adjustment may explain, at least in part, these results.

Our analyses did not support a role for tHcy, cystathionine or tGSH in fat accumulation. Although two meta-analyses reported higher plasma tHcy concentrations in patients with obesity compared to non-obese individuals [10, 11], after controlling for important confounders, tHcy showed inverse or null associations with BMI or fat mass [4–6], in line with current findings. Plasma cystathionine has been positively associated with BMI [4, 12] but showed inconsistent associations with fat mass [5, 6]. Similarly, we found positive associations of cystathionine with BMI and WC in both cohorts, which however did not remain after adjustment for tCys, and inconsistent associations with overall and specific fat depots measures. Finally, our current observations do not corroborate the previously reported inverse association between tGSH and BMI [5, 15], with inverse trends only observed in CODAM after excluding hemolytic samples.

Overall, results suggest that reducing plasma methionine and tCys concentrations via dietary SAA restriction might be a valuable approach for reducing adiposity and improving metabolic health. Dietary restriction of methionine is used as a treatment for human type-1 or classic homocystinuria, a genetic disease characterized by elevated plasma methionine and tHcy and reduced tCys, which causes vascular abnormalities, fatty liver disease and low body fat mass [76, 77]. Improved plasma SAA concentrations in these patients results in reduced fatty liver and increased body fat mass [76, 77], supporting a causal role for methionine and cysteine in body composition in humans. Triangulation of the evidence coming not only from observational studies but also from well-designed randomized controlled trials (RCTs) and genetic studies is required to demonstrate whether lower plasma methionine and tCys concentrations could be effective in preventing or reducing excessive adiposity at a population level. While an ongoing RCT may soon provide important insights [78], to date only a few studies have investigated whether genetic variants associated with plasma SAA concentrations are also associated with obesity, and focused on the methylenetetrahydrofolate reductase gene (MTHFR) C677T polymorphism as determinant of plasma tHcy concentrations [10]. However, these analyses were not able to discriminate whether the causal factor is likely tHcy or one of its metabolic derivatives, and further analyses examining more polymorphisms in relation to all plasma SAA concentrations are needed.

This study has several strengths, including the examination of two independent study populations with similar characteristics and inclusion criteria, in which plasma SAAs were measured following the same protocol. Several measures of overall obesity and specific fat depots were available, enabling a detailed investigation of our research question. Finally, reported estimates had similar strengths in both cohorts and were robust to sensitivity analyses. A few limitations can be also identified. Participants were selected based on underlying health and metabolic risk conditions, including an age range of 40–70 years, which affects the external validity of the findings. Additional research is therefore warranted to examine these associations in other populations. Different assessment methods were used to measure SAT, VAT and liver fat in the two cohorts, and no assessment of body composition was available in CODAM. Despite good reliability has been reported for ultrasound scans [79], MRI is considered among the reference methods to assess abdominal adiposity [79] and is therefore likely to provide more accurate measurements. Furthermore, despite our effort to control for all potential confounders, residual confounding is still possible due to the observational study design. In particular, it was not possible to adjust for plasma vitamins B6, B12 and folate levels, which are known co-factors within the SAA metabolism. Finally, the cross-sectional study design limits any inference of cause–effect relationship between variables.

To conclude, these analyses suggest that plasma methionine and tCys are associated with different patterns of body fat distribution. Plasma methionine concentrations were positively associated with intrahepatic fat deposition and odds of fatty liver disease, while higher plasma tCys concentrations were related to greater general, central and peripheral adiposity. By contrast, no clear relationship emerged between plasma levels of tHcy, cystathionine or tGSH and adiposity measures. Further studies are warranted to obtain a better understanding of the biological processes that underlie these findings, and their potential health consequences.

## Supplementary Information

Below is the link to the electronic supplementary material.

Supplementary file1 (PDF 1443 KB)

## Data Availability

The datasets generated and/or analyzed during the current study are available from the corresponding author and The Maastricht Study Management Team (research.dms@mumc.nl) on reasonable request.
