# Supplementary material for: Associations between plasma sulfur amino acids and specific fat depots in two independent cohorts: CODAM and The Maastricht Study
Source: Eur J Nutr. 2022 Nov 2;62(2):891–904. doi: 10.1007/s00394-022-03041-4 (PMC9941263; doi:10.1007/s00394-022-03041-4)
Supplement: Supplementary file 1 — Supplementary file1 (PDF 1443 KB) [file 394_2022_3041_MOESM1_ESM.pdf]

Associations between plasma sulfur amino acids and specific fat depots in two independent cohorts: CODAM and The Maastricht Study

Elena C. Tore[1,2], Amany K. Elshorbagy[3,4], Frans C.H. Bakers[5], Martijn C.G.J. Brouwers[1,2], Pieter C. Dagnelie[1,2], Simone J.P.M. Eussen[2,6,7], Jacobus F.A. Jansen[5,8], M. Eline Kooi[2,5] , Yvo H.A.M. Kusters[1,2], Steven J.R. Meex[9], Thomas Olsen[10], Helga Refsum[10,3], Kjetil Retterstøl[10], Casper G. Schalkwijk[1,2], Coen D.A. Stehouwer[1,2], Kathrine J. Vinknes[10], Marleen M.J. van Greevenbroek[1,2].

[1]Department of Internal Medicine, Maastricht University, Maastricht, the Netherlands

[2]CARIM School for Cardiovascular Disease, Maastricht University, Maastricht, the Netherlands

[3]Department of Pharmacology, University of Oxford, Oxford, UK

[4]Department of Physiology, Faculty of Medicine, University of Alexandria, Alexandria, Egypt

[5]Department of Radiology & Nuclear Medicine, Maastricht University Medical Center, Maastricht, the Netherlands

[6]Department of Epidemiology, Maastricht University, Maastricht, the Netherlands

[7]CAPHRI Care and Public Health Research Institute, Maastricht University, Maastricht, the Netherlands

[8]MHENS School for Mental Health and Neuroscience, Maastricht University, Maastricht, the Netherlands

[9]Central Diagnostic Laboratory, Maastricht University Medical Center, Maastricht, the Netherlands

[10]Department of Nutrition, Institute of Basic Medical Sciences, University of Oslo, Oslo, Norway

Supplementary material

Figure S1: Graphical representation of the examined associations	3
Figure S2: Crude associations between SAAs and measures of adiposity	4-5
Table S1: Associations between each SAA and measures of obesity and specific fat depots	6-9
Table S2: Model 2 replacing percentage of energy from protein and total calorie intake with total protein intake in grams (sensitivity analyses)	10-11
Table S3: Associations between each SAA and measures of obesity and specific fat depots excluding hemolytic samples (sensitivity analyses)	12-13
Table S4: Associations between each SAA and measures of regional fat distribution adjusted for proxies of muscle mass or measures of prevalent health conditions (sensitivity analyses)	14-17
Table S5: Associations between each SAA and measures of obesity and specific fat depots with additional adjustment for branched-chain amino acids and tyrosine (sensitivity analyses)	18-20
Table S6: Sex-stratified analyses (sensitivity analyses)	21

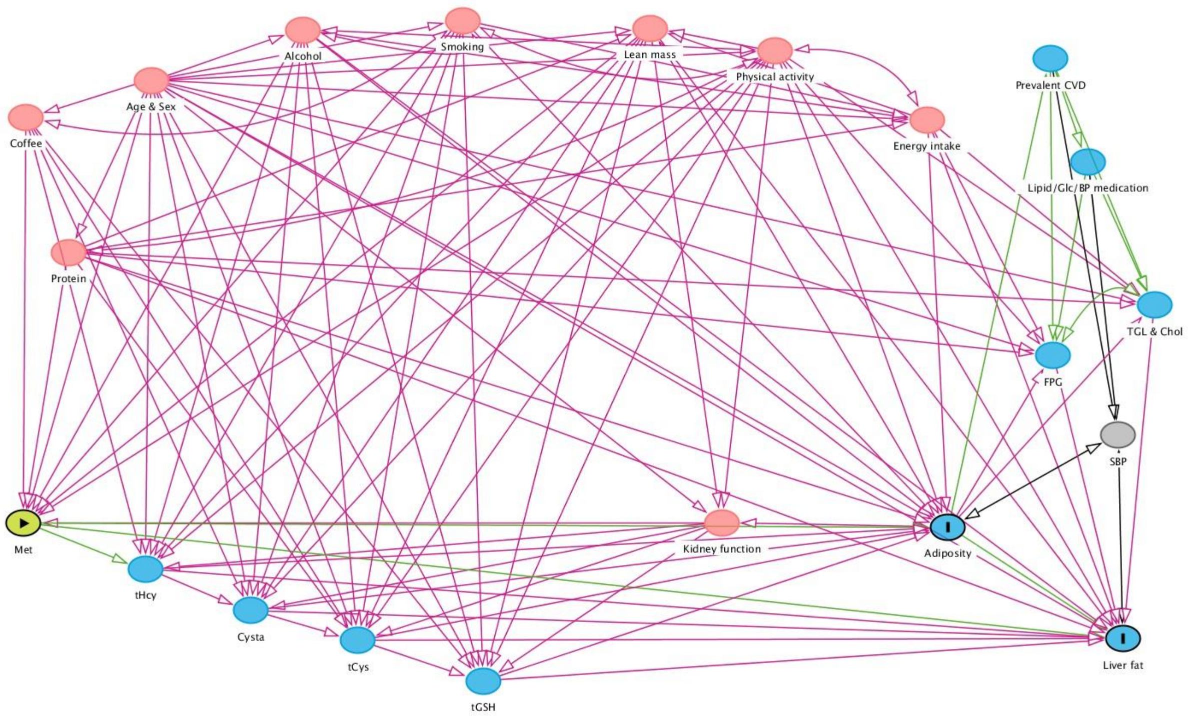

Figure S1: Graphical representation of the examined associations

Note: The association between plasma methionine and measures of adiposity or liver fat. The yellow oval represents the main exposure; blue and black ovals represent the outcomes; blue ovals represent the ancestors of the outcomes; red ovals represent the ancestors of the exposure and the outcome; grey ovals represent any other variable. Green arrows represent causal paths from the exposure; pink arrows represent biasing paths to the exposure and/or the outcome; black arrows represent paths to and from the other variables. Met: plasma methionine; tHcy: plasma total homocysteine; Cysta: plasma cystathionine; tCys: plasma total cysteine; tGSH: plasma total glutathione; protein: dietary protein intake; coffee: coffee consumption; alcohol: alcohol consumption; energy intake: total energy intake; CVD: cardiovascular diseases; lipid/Glc/BP medication: lipid-modifying, glucose-lowering or antihypertensive medications; TGL & Chol: plasma triglycerides and cholesterol; FPG: fasting plasma glucose; SBP: systolic blood pressure.

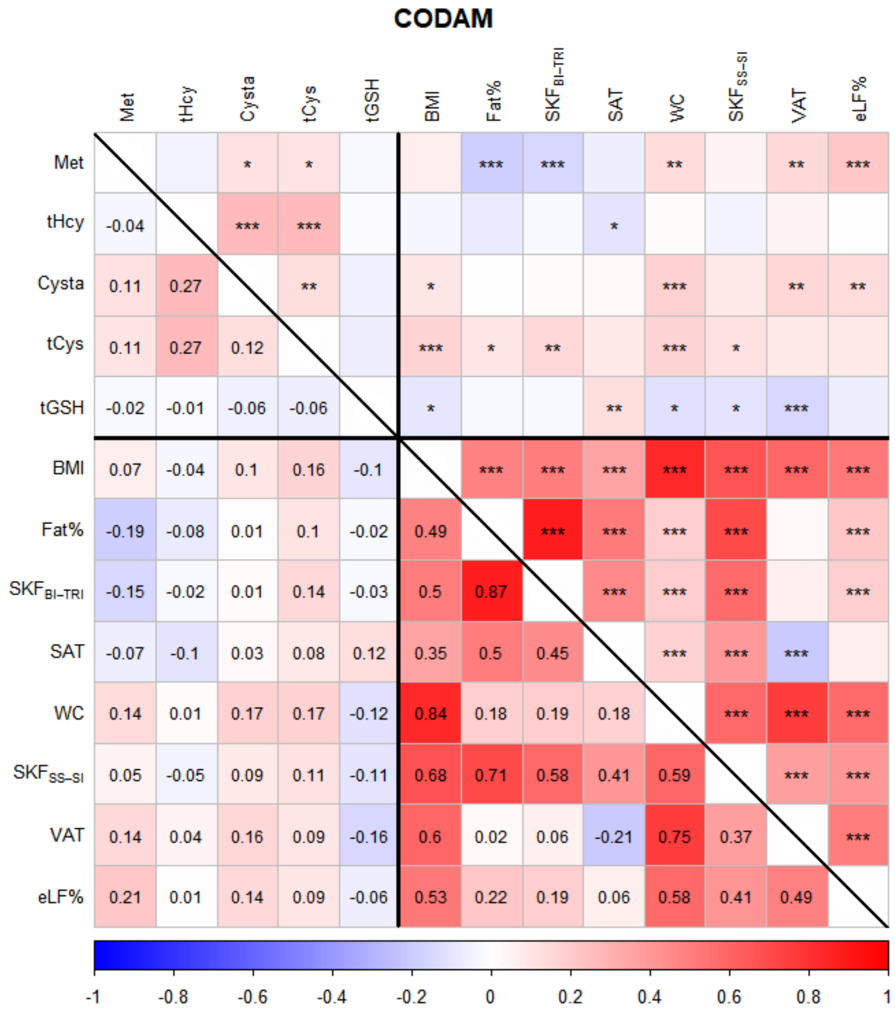

Figure S2A: Crude associations between SAAs and measures of adiposity in CODAM

Beta estimates are reported for the associations between each variable in columns (exposure) and each variable in rows (outcome). CODAM (n=437-470). Met: methionine, tHcy: total homocysteine; Cysta: cystathionine; tCys: total cysteine; tGSH: total glutathione; BMI: body mass index; SKF_{BI-TRI}: sum of bicipital and tricipital skinfolds; SAT: subcutaneous adipose tissue; WC: waist circumference; SKF_{SS-SI}: sum of subscapular and suprailiacal skinfolds; VAT: visceral adipose tissue; eLF%: estimated liver fat percentage. ***: p<0.001, **: p<0.01; *: p<0.05.

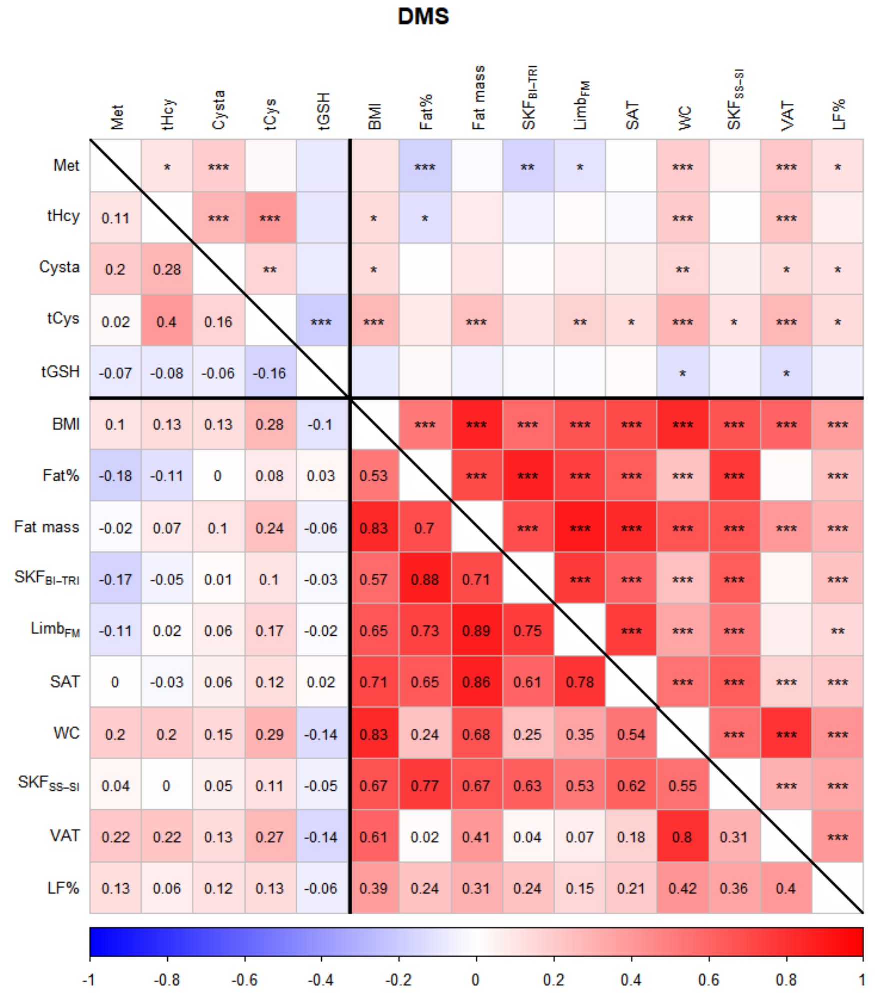

Figure S2B: Crude associations between SAAs and measures of adiposity in DMS

Beta estimates are reported for the associations between each variable in columns (exposure) and each variable in rows (outcome). DMS (N=327-371). Met: methionine, tHcy: total homocysteine; Cysta: cystathionine; tCys: total cysteine; tGSH: total glutathione; BMI: body mass index; SKF_{BI-TRI}: sum of bicipital and tricipital skinfolds; $Limb_{FM}$: limb fat mass; SAT: subcutaneous adipose tissue; WC: waist circumference; SKF_{SS-SI}: sum of subscapular and suprailiacal skinfolds; VAT: visceral adipose tissueLF%: liver fat percentage. ***: p<0.001, **: p<0.01; *: p<0.05.

Table S1: Associations between each SAA and measures of obesity and specific fat depots

	Model	Sample size	Methionine	Total homocysteine	Cystathionine	Total cysteine	Total glutathione
					**β (95% CI)**		
**General obesity**							
**BMI**							
	M1	469*	0.05 (-0.05, 0.14)	-0.05 (-0.14, 0.04)	0.08 (-0.01, 0.17)	**0.18 (0.09, 0.28)**	-0.08 (-0.16, 0.01)
**CODAM**	M2	469*	0.04 (-0.05, 0.14)	-0.05 (-0.14, 0.04)	**0.10 (0.00, 0.19)**	**0.19 (0.09, 0.28)**	-0.07 (-0.16, 0.02)
	M3	468	-0.01 (-0.10, 0.09)	**-0.10 (-0.20, -0.01)**	0.08 (-0.01, 0.18)	**0.20 (0.11, 0.30)**	-0.07 (-0.15, 0.02)
	M1	371	0.08 (-0.02, 0.18)	**0.14 (0.04, 0.24)**	**0.12 (0.03, 0.21)**	**0.27 (0.18, 0.37)**	-0.08 (-0.19, 0.03)
**DMS**	M2	371	0.08 (-0.02, 0.18)	**0.13 (0.03, 0.23)**	**0.10 (0.00, 0.19)**	**0.24 (0.14, 0.34)**	-0.04 (-0.15, 0.07)
	M3	371	0.06 (-0.04, 0.16)	0.06 (-0.05, 0.17)	0.06 (-0.03, 0.16)	**0.22 (0.11, 0.33)**	-0.04 (-0.14, 0.07)
**Fat percentage**							
	M1	463*	0.00 (-0.06, 0.07)	0.00 (-0.06, 0.07)	**0.07 (0.01, 0.13)**	**0.13 (0.06, 0.19)**	**-0.06 (-0.12, -0.00)**
**CODAM**	M2	463*	0.00 (-0.07, 0.07)	0.01 (-0.06, 0.07)	**0.07 (0.01, 0.14)**	**0.13 (0.06, 0.19)**	-0.06 (-0.12, 0.00)
	M3	462	-0.03 (-0.10, 0.03)	-0.03 (-0.10, 0.03)	**0.07 (0.00, 0.13)**	**0.14 (0.07, 0.20)**	-0.05 (-0.11, 0.01)
	M1	368	0.04 (-0.04, 0.11)	**0.08 (0.01, 0.16)**	0.07 (-0.00, 0.14)	**0.18 (0.11, 0.26)**	-0.08 (-0.16, 0.00)
**DMS**	M2	368	0.03 (-0.05, 0.10)	0.07 (-0.00, 0.14)	0.05 (-0.02, 0.12)	**0.17 (0.10, 0.25)**	-0.05 (-0.13, 0.03)
	M3	368	0.02 (-0.05, 0.09)	0.01 (-0.07, 0.09)	0.03 (-0.04, 0.10)	**0.16 (0.08, 0.24)**	-0.05 (-0.13, 0.03)
**Total fat mass**							
	M1	347	0.07 (-0.03, 0.17)	**0.15 (0.05, 0.25)**	**0.11 (0.02, 0.20)**	**0.28 (0.18, 0.38)**	-0.09 (-0.19, 0.02)
**DMS**	M2	347	0.06 (-0.03, 0.16)	**0.14 (0.04, 0.23)**	0.07 (-0.02, 0.17)	**0.23 (0.14, 0.33)**	-0.05 (-0.16, 0.05)
	M3	347	0.05 (-0.05, 0.14)	0.06 (-0.04, 0.17)	0.05 (-0.04, 0.14)	**0.21 (0.10, 0.31)**	-0.05 (-0.15, 0.05)

Table S1 (continued)

	Model	Sample size	Methionine	Total homocysteine	Cystathionine	Total cysteine	Total glutathione
					β (95% CI)		
**Peripheral adiposity**							
**Biceps and triceps skinfolds**							
	M1	464*	-0.00 (-0.08, 0.07)	0.03 (-0.05, 0.11)	0.05 (-0.03, 0.12)	**0.15 (0.07, 0.23)**	-0.06 (-0.13, 0.01)
**CODAM**	M2	464*	0.00 (-0.08, 0.08)	0.03 (-0.05, 0.11)	0.04 (-0.04, 0.12)	**0.15 (0.08, 0.23)**	-0.05 (0.12, 0.02)
	M3	463	-0.03 (-0.11, 0.05)	-0.02 (-0.11, 0.06)	0.04 (-0.03, 0.12)	**0.16 (0.08, 0.24)**	-0.05 (-0.12, 0.03)
	M1	368	0.00 (-0.09, 0.09)	**0.13 (0.04, 0.21)**	0.06 (-0.02, 0.15)	**0.21 (0.12, 0.29)**	**-0.12 (-0.22, -0.02)**
**DMS**	M2	368	-0.00 (-0.09, 0.09)	**0.13 (0.04, 0.22)**	0.03 (-0.05, 0.11)	**0.20 (0.12, 0.29)**	-0.09 (-0.18, 0.01)
	M3	368	-0.01 (-0.10, 0.08)	0.07 (-0.03, 0.16)	0.02 (-0.06, 0.10)	**0.17 (0.07, 0.26)**	-0.08 (-0.18, 0.01)
**Limb fat mass**							
	M1	347	0.06 (-0.03, 0.15)	**0.16 (0.07, 0.25)**	**0.11 (0.02, 0.19)**	**0.25 (0.16, 0.34)**	-0.10 (-0.20, 0.00)
**DMS**	M2	347	0.05 (-0.04, 0.15)	**0.15 (0.06, 0.24)**	0.06 (-0.03, 0.15)	**0.22 (0.13, 0.31)**	-0.07 (-0.16, 0.03)
	M3	347	0.05 (-0.05, 0.13)	0.08 (-0.02, 0.18)	0.04 (-0.05, 0.13)	**0.18 (0.08, 0.28)**	-0.06 (-0.16, 0.04)
**Subcutaneous trunk adipose tissue**							
**SAT**							
	M1	437*	0.05 (-0.04, 0.14)	-0.03 (-0.12, 0.06)	**0.10 (0.01, 0.18)**	**0.14 (0.05, 0.23)**	**0.09 (0.01, 0.18)**
**CODAM (US)**	M2	437*	0.04 (-0.05, 0.14)	-0.04 (-0.13, 0.05)	**0.09 (0.00, 0.19)**	**0.14 (0.05, 0.23)**	**0.09 (0.01, 0.18)**
	M3	436	0.00 (-0.09, 0.10)	-0.08 (-0.17, 0.01)	0.09 (-0.00, 0.18)	**0.16 (0.07, 0.26)**	**0.10 (0.01, 0.18)**
	M1	370	0.09 (-0.01, 0.20)	0.07 (-0.03, 0.17)	0.08 (-0.01, 0.18)	**0.19 (0.09, 0.30)**	-0.02 (-0.14, 0.09)
**DMS (MRI)**	M2	370	0.08 (-0.02, 0.18)	0.06 (-0.04, 0.16)	0.06 (-0.04, 0.16)	**0.18 (0.07, 0.28)**	0.01 (-0.10, 0.12)
	M3	370	0.07 (-0.03, 0.17)	-0.01 (-0.12, 0.10)	0.04 (-0.06, 0.14)	**0.18 (0.07, 0.29)**	0.02 (-0.10, 0.13)

Table S1 (continued)

	Model	Sample size	Methionine	Total homocysteine	Cystathionine	Total cysteine	Total glutathione
					**β (95% CI)**		
**Central adiposity**							
**Waist**							
	M1	468*	0.01 (-0.08, 0.10)	-0.05 (-0.14, 0.04)	**0.12 (0.04, 0.21)**	**0.17 (0.09, 0.26)**	-0.07(-0.15, 0.01)
**CODAM**	M2	468*	0.01 (-0.08, 0.10)	-0.05 (-0.13, 0.04)	**0.14 (0.06, 0.23)**	**0.16 (0.07, 0.25)**	-0.06 (-0.14, 0.03)
	M3	467	-0.05 (-0.13, 0.04)	**-0.11 (-0.20, -0.02)**	**0.14 (0.05, 0.23)**	**0.19 (0.10, 0.28)**	-0.05 (-0.13, 0.03)
	M1	371	0.07 (-0.03, 0.16)	**0.10 (0.01, 0.19)**	**0.10 (0.01, 0.18)**	**0.20 (0.11, 0.29)**	-0.05 (-0.15, 0.06)
**DMS**	M2	371	0.06 (-0.03, 0.15)	**0.09 (0.00, 0.18)**	**0.09 (0.00, 0.18)**	**0.17 (0.08, 0.27)**	-0.02 (-0.13, 0.08)
	M3	371	0.04 (-0.05, 0.14)	0.02 (-0.08, 0.12)	0.07 (-0.02, 0.16)	**0.16 (0.06, 0.26)**	-0.02 (-0.12, 0.08)
**Suprailiacal and subscapular skinfolds**							
	M1	463*	0.04 (-0.05, 0.14)	-0.02 (-0.11, 0.07)	**0.10 (0.01, 0.19)**	**0.17 (0.07, 0.26)**	**-0.09 (-0.18, -0.00)**
**CODAM**	M2	463*	0.03 (-0.07, 0.12)	-0.01 (-0.10, 0.08)	**0.11 (0.02, 0.20)**	**0.17 (0.08, 0.27)**	**-0.09 (-0.17, -0.00)**
	M3	462	-0.01 (-0.11, 0.08)	-0.06 (-0.15. 0.04)	**0.10 (0.00, 0.19)**	**0.18 (0.08, 0.28)**	-0.08 (-0.17, 0.00)
	M1	368	0.07 (-0.03, 0.18)	0.08 (-0.02, 0.17)	0.08 (-0.01, 0.17)	**0.18 (0.08, 0.28)**	-0.08 (-0.19, 0.04)
**DMS**	M2	368	0.06 (-0.04, 0.16)	0.05 (-0.04, 0.15)	0.06 (-0.03, 0.16)	**0.16 (0.06, 0.26)**	-0.05 (-0.16, 0.06)
	M3	368	0.05 (-0.05, 0.15)	-0.00 (-0.11, 0.11)	0.04 (-0.06, 0.14)	**0.15 (0.04, 0.26)**	-0.05 (-0.16, 0.06)
**VAT**							
	M1	439*	0.00 (-0.09, 0.09)	-0.03 (-0.12, 0.05)	0.09 (-0.00, 0.17)	0.05 (-0.04, 0.14)	**-0.10 (-0.18, -0.02)**
**CODAM (US)**	M2	439*	0.00 (-0.09, 0.09)	-0.03 (-0.12, 0.05)	**0.10 (0.01, 0.19)**	0.05 (-0.04, 0.14)	**-0.10 (-0.18, -0.02)**
	M3	438	-0.02 (-0.12, 0.07)	-0.06 (-0.15, 0.03)	**0.10 (0.00, 0.19)**	0.06 (-0.03, 0.15)	**-0.09 (-0.18, -0.01)**
	M1	369	0.05 (-0.04, 0.13)	0.07 (-0.01, 0.16)	0.07 (-0.01, 0.15)	**0.14 (0.05, 0.23)**	-0.03 (-0.12, 0.07)
**DMS (MRI)**	M2	369	0.06 (-0.03, 0.14)	**0.08 (0.00, 0.17)**	0.08 (-0.01, 0.18)	**0.12 (0.03, 0.20)**	-0.01 (-0.11, 0.08)
	M3	369	0.04 (-0.04, 0.13)	0.04 (-0.06, 0.13)	0.05 (-0.03, 0.14)	**0.10 (0.01, 0.20)**	-0.01 (-0.11, 0.09)

Table S1 (continued)

	Model	Sample size	Methionine	Total homocysteine	Cystathionine	Total cysteine	Total glutathione
					**β (95% CI)**		
**Fatty liver**							
**Estimated liver fat %**							
	M1	465*	**0.12 (0.04, 0.20)**	-0.03 (-0.11, 0.05)	**0.10 (0.02, 0.17)**	**0.10 (0.02, 0.18)**	0.00 (-0.08, 0.07)
**CODAM**	M2	465*	**0.12 (0.04, 0.20)**	-0.03 (-0.11, 0.05)	**0.10 (0.02, 0.18)**	**0.10 (0.02, 0.18)**	0.00 (-0.08, 0.08)
	M3	464	**0.09 (0.01, 0.16)**	-0.03 (-0.10, 0.05)	0.06 (-0.01, 0.13)	0.02 (-0.05, 0.10)	0.03 (-0.04, 0.09)
**Liver fat %**							
	M1	351	**0.13 (0.01, 0.26)**	0.06 (-0.05, 0.17)	**0.11 (0.01, 0.21)**	**0.13 (0.02, 0.24)**	-0.04 (-0.16, 0.08)
**DMS**	M2	351	**0.14 (0.02, 0.27)**	0.08 (-0.03, 0.19)	**0.12 (0.01, 0.22)**	0.08 (-0.03, 0.19)	-0.02 (-0.14, 0.10)
	M3	351	0.12 (-0.00, 0.24)	0.02 (-0.10, 0.13)	0.08 (-0.02, 0.18)	-0.01 (-0.12, 0.11)	0.00 (-0.12, 0.11)
					**OR (95% CI)**		
**Fatty liver**							
	M1	420*	**1.50 (1.20, 1.88)**	0.95 (0.77, 1.16)	0.96 (0.78, 1.18)	1.14 (0.92, 1.42)	1.10 (0.91, 1.36)
**CODAM**	M2	420*	**1.49 (1.19, 1.88)**	0.97 (0.79, 1.19)	0.97 (0.78, 1.20)	1.14 (0.92, 1.43)	1.10 (0.91, 1.36)
	M3	419	**1.51 (1.20, 1.92)**	1.01 (0.81, 1.27)	0.87 (0.68, 1.10)	0.97 (0.76, 1.24)	1.13 (0.93, 1.41)
	M1	351	**1.40 (1.03, 1.94)**	1.16 (0.91, 1.48)	**1.27 (1.01, 1.60)**	**1.43 (1.11, 1.85)**	0.72 (0.39, 1.08)
**DMS**	M2	351	**1.51 (1.09, 2.14)**	1.23 (0.95, 1.59)	**1.31 (1.03, 1.68)**	**1.31 (1.01, 1.72)**	0.80 (0.44, 1.17)
	M3	351	**1.60 (1.11, 2.35)**	1.10 (0.80, 1.49)	1.21 (0.92, 1.58)	1.04 (0.76, 1.42)	0.88 (0.47, 1.30)

Standardized beta coefficients (95% confidence intervals) are shown. M1: adjusted for age, sex, glucose metabolism status; M2 (main model): adjusted for M1 plus smoking status, alcohol and coffee consumption, physical activity, progenitor SAA, protein intake and total energy consumption (in models with plasma methionine or total cysteine as main exposure), height (in models with DXA-derived measures as main outcome) and, were applicable, for the lag-time before MRI and DXA scans; M3: adjusted for M2 plus all plasma SAAs and protein intake and total energy consumption if not already adjusted for in M2. * In models with cystathionine, one participant was excluded because of an extreme value. Figures in bold represent statistically significant associations (p<0.05).

Table S2: Model 2 replacing percentage of energy from protein and total calorie intake with total protein intake in grams (sensitivity analyses)

	Model	Sample size	Methionine	Total cysteine
			β (95% CI)	
**General obesity**				
**BMI**				
**CODAM**	M2a	469	0.05 (-0.05, 0.14)	**0.18 (0.08, 0.27)**
**DMS**	M2a	351	0.08 (-0.02, 0.18)	**0.25 (0.15, 0.35)**
**Fat percentage**				
**CODAM**	M2a	463	0.00 (-0.06, 0.07)	**0.13 (0.07, 0.20)**
**DMS**	M2a	368	0.03 (-0.05, 0.10)	**0.17 (0.10, 0.25)**
**Total fat mass**				
**DMS**	M2a	347	0.07 (-0.04, 0.16)	**0.24 (0.14, 0.33)**
**Peripheral adiposity**				
**Biceps and triceps skinfolds**				
**CODAM**	M2a	464	-0.00 (-0.08, 0.08)	**0.16 (0.08, 0.24)**
**DMS**	M2a	368	-0.00 (-0.09, 0.09)	**0.20 (0.12, 0.29)**
**Limb fat mass**				
**DMS**	M2a	347	0.05 (-0.04, 0.15)	**0.22 (0.13, 0.31)**
**Subcutaneous trunk adipose tissue**				
**SAT**				
**CODAM (US)**	M2a	437	0.05 (-0.05, 0.14)	**0.13 (0.04, 0.23)**
**DMS (MRI)**	M2a	370	0.08 (-0.02, 0.18)	**0.18 (0.07, 0.28)**
**Central adipose tissue**				
**Waist**				
**CODAM**	M2a	468	0.01 (-0.08, 0.10)	**0.17 (0.08, 0.26)**
**DMS**	M2a	372	0.06 (-0.03, 0.15)	**0.17 (0.08, 0.27)**
**Suprailiacal and subscapular skinfolds**				
**CODAM**	M2a	463	0.04 (-0.06, 0.13)	**0.17 (0.07, 0.26)**
**DMS**	M2a	368	0.06 (-0.04, 0.16)	**0.16 (0.06, 0.26)**
**VAT**				
**CODAM (US)**	M2a	441	0.00 (-0.09, 0.09)	0.05 (-0.04, 0.14)
**DMS (MRI)**	M2a	369	0.06 (-0.03, 0.14)	**0.12 (0.03, 0.20)**

Table S2 (continued)

	Model	Sample size	Methionine	Total cysteine
			β (95% CI)	
**Liver fat**				
**Estimated liver fat %**				
**CODAM**	M2a	465	**0.12 (0.04, 0.20)**	**0.09 (0.00, 0.17)**
**Liver fat %**				
**DMS**	M2a	351	**0.15 (0.02, 0.27)**	0.08 (-0.03, 0.19)
			OR (95% CI)	
**Fatty liver**				
**CODAM**	M2a	420	**1.48 (1.19, 1.87)**	1.07 (0.85, 1.33)
**DMS**	M2a	351	**1.51 (1.10, 2.13)**	**1.31 (1.01, 1.72)**

Standardized beta coefficients (95% confidence intervals) are shown. Adjusted for age, sex, glucose metabolism status, smoking status, alcohol and coffee consumption, physical activity, progenitor SAA, protein intake, height (in models with DXA-derived measures as main outcome) and, were applicable, for the lag-time before MRI and DXA scans. * In models with cystathionine, one participant was excluded because of an extreme value. Figures in bold represent statistically significant associations ($p<0.05$).

Table S3: Associations between each SAA and measures of obesity and specific fat depots excluding hemolytic samples (sensitivity analyses)

	Model	Sample size	Methionine	Total homocysteine	Cystathionine	Total cysteine	Total glutathione
					**β (95% CI)**		
**General obesity**							
**BMI**							
**CODAM**	M2b	418*	0.05 (-0.05, 0.15)	-0.07 (-0.16, 0.03)	**0.10 (0.00, 0.20)**	**0.16 (0.06, 0.26)**	**-0.15 (-0.24, -0.06)**
**DMS**	M2b	313	0.04 (-0.06, 0.15)	**0.12 (0.02, 0.23)**	0.09 (-0.01, 0.19)	**0.23 (0.13, 0.34)**	-0.06 (-0.15, 0.04)
**Fat percentage**							
**CODAM**	M2b	412*	-0.01 (-0.08, 0.06)	0.00 (-0.07, 0.07)	**0.07 (0.01, 0.14)**	**0.11 (0.04, 0.18)**	**-0.06 (-0.13, -0.00)**
**DMS**	M2b	327	0.01 (-0.07, 0.09)	0.07 (-0.01, 0.14)	0.04 (-0.04, 0.11)	**0.16 (0.08, 0.24)**	0.02 (-0.10, 0.05)
**Total fat mass**							
**DMS**	M2b	308	0.03 (-0.07, 0.13)	**0.13 (0.03, 0.23)**	0.07 (-0.03, 0.17)	**0.21 (0.10, 0.31)**	0.01 (-0.09, 0.10)
**Peripheral adipose tissue**							
**Biceps and triceps skinfolds**							
**CODAM**	M2b	413*	-0.00 (-0.09, 0.08)	0.02 (-0.07, 0.10)	0.05 (-0.03, 0.13)	**0.14 (0.05, 0.22)**	**-0.08 (-0.16, -0.00)**
**DMS**	M2d	327	0.00 (-0.09, 0.10)	**0.12 (0.02, 0.21)**	0.03 (-0.06, 0.12)	**0.20 (0.10, 0.29)**	-0.06 (-0.15, 0.02)
**Limb fat mass**							
**DMS**	M2b	308	0.03 (-0.07, 0.12)	**0.14 (0.04, 0.23)**	0.06 (-0.04, 0.15)	**0.20 (0.10, 0.29)**	-0.01 (-0.10, 0.08)
**Subcutaneous adipose tissue**							
**SAT**							
**CODAM (US)**	M2b	391*	0.04 (-0.06, 0.14)	-0.03 (-0.12, 0.07)	0.09 (-0.01, 0.19)	**0.15 (0.05, 0.25)**	-0.03 (-0.12, 0.06)
**DMS (MRI)**	M2b	328	0.03 (-0.08, 0.14)	0.08 (-0.03, 0.18)	0.05 (-0.05, 0.15)	**0.17 (0.06, 0.28)**	-0.00 (-0.10, 0.10)

Table S3 (continued)

	Model	Sample size	Methionine	Total homocysteine	Cystathionine	Total cysteine	Total glutathione
					**β (95% CI)**		
**Central adiposity**							
**Waist**							
**CODAM**	M2b	417*	0.01 (-0.09, 0.11)	-0.05 (-0.15, 0.04)	**0.10 (0.00, 0.20)**	**0.14 (0.04, 0.23)**	**-0.15 (-0.23, -0.06)**
**DMS**	M2b	330	0.02 (-0.08, 0.12)	0.08 (-0.01, 0.18)	0.08 (-0.01, 0.17)	**0.17 (0.07, 0.26)**	-0.05 (-0.14, 0.04)
**Suprailiacal and subscapular skinfolds**							
**CODAM**	M2b	412*	0.01 (-0.09, 0.11)	-0.02 (-0.12, 0.08)	**0.10 (0.01, 0.20)**	**0.14 (0.04, 0.24)**	-0.08 (-0.17, 0.02)
**DMS**	M2b	327	0.04 (-0.07, 0.14)	0.05 (-0.05, 0.16)	0.04 (-0.06, 0.14)	**0.14 (0.03, 0.24)**	-0.01 (-0.10, 0.09)
**VAT**							
**CODAM (US)**	M2b	392*	-0.01 (-0.10, 0.09)	-0.04 (-0.13, 0.05)	**0.10 (0.01, 0.20)**	0.03 (-0.07, 0.12)	**-0.14 (-0.23, -0.05)**
**DMS (MRI)**	M2b	327	0.05 (-0.05, 0.14)	0.08 (-0.01, 0.17)	0.07 (-0.02, 0.15)	**0.11 (0.02, 0.21)**	0.01 (-0.07, 0.10)
**Fatty liver**							
**Estimated liver fat %**							
**CODAM**	M2b	419*	**0.14 (0.04, 0.25)**	-0.03 (-0.11, 0.05)	**0.10 (0.01, 0.18)**	**0.11 (0.02, 0.19)**	-0.04 (-0.12, 0.04)
**Liver fat %**							
**DMS**	M2b	313	**0.13 (0.01, 0.26)**	0.07 (-0.05, 0.18)	**0.13 (0.02, 0.24)**	0.08 (-0.04, 0.20)	-0.06 (-0.17, 0.05)
					**OR (95% CI)**		
**Fatty liver**							
**CODAM**	M2b	373*	**1.50 (1.19, 1.93)**	0.97 (0.78, 1.20)	0.94 (0.75, 1.18)	1.12 (0.89, 1.41)	0.99 (0.79, 1.22)
**DMS**	M2b	313	1.34 (0.97, 1.90)	1.19 (0.91, 1.56)	**1.37 (1.07, 1.78)**	1.27 (0.95, 1.69)	0.66 (0.34, 1.10)

Standardized beta coefficients (95% confidence intervals) are shown. Adjusted for age, sex, glucose metabolism status, smoking status, alcohol and coffee consumption, physical activity, progenitor SAA, protein intake and total energy consumption (in models with plasma methionine or total cysteine as main exposure), height (in models with DXA-derived measures as main outcome) and, were applicable, for the lag-time before MRI and DXA scans. * In models with cystathionine, one participant was excluded because of an extreme value. Figures in bold represent statistically significant associations (p<0.05).

Table S4: Associations between each SAA and measures of regional fat distribution adjusted for proxies of muscle mass or measures of prevalent health conditions (sensitivity analyses)

	Model	Sample size	Methionine	Total homocysteine	Cystathionine	Total cysteine	Total glutathione
**General obesity**					β (95% CI)		
**BMI**							
**CODAM**	M2c	467*	0.05 (-0.05, 0.15)	-0.04 (-0.14, 0.06)	**0.12 (0.02, 0.22)**	**0.22 (0.13, 0.32)**	-0.06 (-0.15, 0.03)
	M2f	439*	0.06 (-0.03, 0.16)	-0.08 (-0.18, 0.01)	**0.12 (0.02, 0.21)**	**0.20 (0.11, 0.30)**	-0.10 (-0.21, 0.01)
	M2g	435*	0.01 (-0.07, 0.09)	-0.06 (-0.14, 0.02)	0.00 (-0.08, 0.08)	**0.14 (0.06, 0.22)**	-0.01 (-0.08, 0.06)
**DMS**	M2c	371	0.05 (-0.05, 0.15)	0.10 (-0.01, 0.20)	0.08 (-0.01, 0.18)	**0.22 (0.12, 0.32)**	-0.05 (-0.16, 0.06)
	M2d	347	0.07 (-0.01, 0.15)	0.08 (-0.01, 0.16)	0.05 (-0.03, 0.13)	**0.11 (0.02, 0.20)**	-0.07 (-0.16, 0.02)
	M2e	347	0.09 (-0.01, 0.19)	**0.13 (0.03, 0.24)**	0.09 (-0.01, 0.19)	**0.23 (0.13, 0.34)**	-0.04 (-0.15, 0.07)
	M2f	349	0.01 (-0.09, 0.12)	**0.11 (0.00, 0.21)**	0.05 (-0.04, 0.15)	**0.20 (0.10, 0.30)**	-0.02 (-0.13, 0.08)
**Fat percentage**							
**CODAM**	M2c	461*	-0.00 (-0.07, 0.06)	-0.00 (-0.07, 0.06)	**0.08 (0.01, 0.14)**	**0.13 (0.06, 0.20)**	-0.05 (-0.11, 0.01)
	M2f	437*	0.01 (-0.05, 0.08)	-0.02 (-0.08, 0.05)	**0.08 (0.02, 0.15)**	**0.11 (0.04, 0.18)**	**-0.08 (-0.16, -0.00)**
	M2g	430*	-0.00 (-0.07, 0.06)	0.00 (-0.06, 0.06)	0.02 (-0.04, 0.09)	**0.10 (0.04, 0.16)**	-0.03 (-0.09, 0.02)
**DMS**	M2c	368	0.01 (-0.07, 0.09)	0.05 (-0.03, 0.12)	0.04 (-0.03, 0.11)	**0.16 (0.08, 0.24)**	-0.06 (-0.14, 0.02)
	M2d	344	0.03 (-0.04, 0.09)	0.05 (-0.02, 0.12)	0.03 (-0.04, 0.10)	**0.11 (0.04, 0.18)**	-0.06 (-0.14, 0.01)
	M2e	344	0.03 (-0.04, 0.11)	**0.08 (0.00, 0.15)**	0.05 (-0.02, 0.12)	**0.16 (0.09, 0.24)**	-0.05 (-0.13, 0.03)
	M2f	365	-0.02 (-0.10, 0.06)	0.05 (-0.03, 0.12)	0.02 (-0.05, 0.09)	**0.14 (0.06, 0.21)**	-0.04 (-0.12, 0.04)
**Total fat mass**							
**DMS**	M2c	347	0.04 (-0.06, 0.14)	**0.11 (0.01, 0.21)**	0.06 (-0.03, 0.16)	**0.22 (0.12, 0.32)**	-0.06 (-0.17, 0.04)
	M2d	347	0.06 (-0.02, 0.13)	**0.10 (0.02, 0.17)**	0.04 (-0.03, 0.11)	**0.13 (0.05, 0.21)**	-0.05 (-0.13, 0.03)
	M2e	347	0.07 (-0.03, 0.17)	**0.15 (0.05, 0.25)**	0.07 (-0.02, 0.17)	**0.26 (0.16, 0.35)**	-0.05 (-0.16, 0.05)
	M2f	344	0.02 (-0.09, 0.12)	**0.12 (0.02, 0.22)**	0.05 (-0.05, 0.14)	**0.20 (0.10, 0.30)**	-0.05 (-0.15, 0.06)

Table S4 (continued)

	Model	Sample size	Methionine	Total homocysteine	Cystathionine	Total cysteine	Total glutathione
**Peripheral adiposity**					β (95% CI)		
**Biceps and triceps skinfolds**							
**CODAM**	M2c	462*	0.00 (-0.08, 0.08)	0.03 (-0.06, 0.11)	0.05 (-0.03, 0.13)	**0.17 (0.08, 0.25)**	-0.04 (-0.12, 0.03)
	M2f	438*	0.02 (-0.07, 0.10)	0.01 (-0.07, 0.09)	0.07 (-0.01, 0.15)	**0.15 (0.07, 0.24)**	-0.06 (-0.15, 0.03)
	M2g	431*	0.00 (-0.07, 0.08)	0.03 (-0.05, 0.10)	0.00 (-0.08, 0.08)	**0.13 (0.06, 0.21)**	-0.03 (-0.10, 0.04)
**DMS**	M2c	368	-0.02 (-0.11, 0.07)	**0.11 (0.02, 0.20)**	0.03 (-0.06, 0.11)	**0.20 (0.10, 0.29)**	-0.09 (-0.19, 0.01)
	M2d	344	-0.01 (-0.09, 0.08)	**0.12 (0.03, 0.20)**	0.01 (-0.07, 0.09)	**0.15 (0.06, 0.24)**	-0.09 (-0.18, 0.00)
	M2e	344	0.00 (-0.09, 0.09)	**0.14 (0.05, 0.23)**	0.03 (-0.06, 0.12)	**0.21 (0.11, 0.30)**	-0.08 (-0.18, 0.01)
	M2f	365	-0.02 (-0.12, 0.07)	**0.12 (0.03, 0.21)**	0.01 (-0.08, 0.09)	**0.18 (0.09, 0.27)**	-0.08 (-0.17, 0.02)
**Limb fat mass**							
**DMS**	M2c	347	0.03 (-0.06, 0.13)	**0.12 (0.03, 0.22)**	0.05 (-0.03, 0.14)	**0.21 (0.11, 0.30)**	-0.07 (-0.17, 0.02)
	M2d	347	0.05 (-0.03, 0.13)	**0.12 (0.04, 0.20)**	0.03 (-0.04, 0.11)	**0.14 (0.06, 0.22)**	-0.06 (-0.15, 0.02)
	M2e	347	0.06 (-0.04, 0.15)	**0.16 (0.06, 0.25)**	0.06 (-0.03, 0.15)	**0.24 (0.14, 0.33)**	-0.07 (-0.16, 0.03)
	M2f	344	0.02 (-0.08, 0.12)	**0.13 (0.03, 0.22)**	0.04 (-0.05, 0.13)	**0.21 (0.12, 0.31)**	-0.07 (-0.17, 0.03)
**Subcutaneous adipose tissue**							
**SAT**							
**CODAM (US)**	M2c	435*	0.04 (-0.05, 0.13)	-0.05 (-0.14, 0.05)	0.09 (-0.00, 0.19)	**0.15 (0.05, 0.25)**	**0.10 (0.02, 0.18)**
	M2f	425*	0.04 (-0.06, 0.13)	-0.05 (-0.14, 0.05)	0.08 (-0.02, 0.17)	**0.14 (0.05, 0.24)**	0.07 (-0.04, 0.19)
	M2g	409*	0.02 (-0.08, 0.11)	-0.01 (-0.10, 0.08)	0.05 (-0.05, 0.14)	**0.10 (0.01, 0.20)**	**0.10 (0.02, 0.18)**
**DMS (MRI)**	M2c	370	0.06 (-0.04, 0.17)	0.04 (-0.07, 0.14)	0.05 (-0.04, 0.15)	**0.16 (0.06, 0.27)**	0.01 (-0.11, 0.12)
	M2d	346	0.07 (-0.02, 0.17)	0.01 (-0.08, 0.10)	0.03 (-0.07, 0.12)	0.07 (-0.03, 0.17)	-0.01 (-0.11, 0.09)
	M2e	346	0.09 (-0.02, 0.19)	0.05 (-0.06, 0.16)	0.06 (-0.05, 0.16)	**0.17 (0.06, 0.27)**	0.01 (-0.11, 0.12)
	M2f	367	0.03 (-0.08, 0.15)	**0.02 (-0.09, 0.13)**	0.04 (-0.06, 0.14)	**0.14 (0.03, 0.25)**	0.01 (-0.10, 0.13)

Table S4 (continued)

	Model	Sample size	Methionine	Total homocysteine	Cystathionine	Total cysteine	Total glutathione
**Central adiposity**					**β (95% CI)**		
**Waist**							
	M2c	466*	0.00 (-0.09, 0.10)	-0.06 (-0.15, 0.04)	**0.15 (0.06, 0.25)**	**0.19 (0.09, 0.28)**	-0.05 (-0.13, 0.03)
**CODAM**	M2f	439*	0.01 (-0.08, 0.11)	-0.09 (-0.18, 0.00)	**0.12 (0.02, 0.21)**	**0.17 (0.07, 0.26)**	-0.10 (-0.21, 0.00)
	M2g	434*	-0.02 (-0.09, 0.06)	-0.06 (-0.14, 0.02)	0.06 (-0.02, 0.13)	**0.12 (0.05, 0.20)**	-0.00 (-0.07, 0.06)
	M2c	371	0.03 (-0.06, 0.12)	0.05 (-0.05, 0.14)	0.08 (-0.01, 0.16)	**0.15 (0.05, 0.24)**	-0.03 (-0.13, 0.07)
	M2d	347	0.05 (-0.03, 0.12)	0.03 (-0.04, 0.11)	0.05 (-0.03, 0.12)	0.04 (-0.04, 0.12)	-0.05 (-0.13, 0.03)
**DMS**	M2e	347	0.06 (-0.03, 0.16)	0.09 (-0.01, 0.18)	0.08 (-0.01, 0.18)	**0.16 (0.06, 0.26)**	-0.02 (-0.13, 0.08)
	M2f	369	-0.01 (-0.11, 0.08)	0.05 (-0.04, 0.14)	0.05 (-0.04, 0.13)	**0.11 (0.02, 0.20)**	0.00 (-0.10, 0.10)
**Suprailiacal and subscapular skinfolds**							
	M2c	461*	0.03 (-0.07, 0.12)	-0.02 (-0.12, 0.08)	**0.12 (0.02, 0.21)**	**0.18 (0.08, 0.28)**	-0.08 (-0.17, 0.01)
**CODAM**	M2f	437*	0.05 (-0.04, 0.15)	-0.04 (-0.14, 0.05)	**0.12 (0.03, 0.22)**	**0.15 (0.05, 0.24)**	**-0.11 (-0.22, -0.00)**
	M2g	430*	0.02 (-0.07, 0.11)	-0.03 (-0.11, 0.06)	0.04 (-0.05, 0.13)	**0.13 (0.04, 0.22)**	-0.05 (-0.13, 0.03)
	M2c	368	0.04 (-0.06, 0.15)	0.03 (-0.07, 0.14)	0.06 (-0.04, 0.15)	**0.15 (0.05, 0.25)**	-0.06 (-0.17, 0.05)
	M2d	344	0.06 (-0.04, 0.15)	0.01 (-0.09, 0.10)	0.05 (-0.04, 0.14)	0.07 (-0.03, 0.17)	-0.07 (-0.17, 0.03)
**DMS**	M2e	344	0.07 (-0.04, 0.17)	0.04 (-0.06, 0.15)	0.08 (-0.02, 0.18)	**0.15 (0.04, 0.25)**	-0.06 (-0.17, 0.06)
	M2f	365	0.01 (-0.10, 0.12)	0.03 (-0.08, 0.13)	0.03 (-0.07, 0.12)	**0.11 (0.01, 0.21)**	-0.03 (-0.14, 0.08)
**VAT**							
	M2c	437*	-0.00 (-0.09, 0.09)	-0.04 (-0.14, 0.05)	**0.11 (0.02, 0.20)**	0.06 (-0.04, 0.15)	**-0.09 (-0.18, -0.01)**
**CODAM (US)**	M2f	427*	0.00 (-0.09, 0.09)	-0.07 (-0.16, 0.02)	**0.09 (0.01, 0.18)**	0.02 (-0.08, 0.11)	**-0.14 (-0.25, -0.04)**
	M2g	410*	-0.03 (-0.11, 0.05)	-0.05 (-0.13, 0.03)	0.03 (-0.05, 0.12)	0.00 (-0.08, 0.08)	-0.05 (-0.12, 0.02)
	M2c	369	0.04 (-0.05, 0.12)	0.06 (-0.03, 0.15)	0.06 (-0.02, 0.14)	**0.10 (0.01, 0.19)**	-0.02 (-0.12, 0.07)
	M2d	345	0.04 (-0.04, 0.12)	0.05 (-0.03, 0.13)	0.04 (-0.03, 0.12)	0.04 (-0.05, 0.12)	-0.03 (-0.12, 0.06)
**DMS (MRI)**	M2e	345	0.05 (-0.04, 0.14)	0.08 (-0.00, 0.17)	0.08 (-0.02, 0.15)	**0.11 (0.02, 0.20)**	-0.02 (-0.11, 0.08)
	M2f	366	0.00 (-0.09, 0.09)	**0.08 (0.00, 0.17)**	0.03 (-0.05, 0.11)	0.06 (-0.03, 0.15)	0.01 (-0.08, 0.10)

Table S4 (continued)

	Model	Sample size	Methionine	Total homocysteine	Cystathionine	Total cysteine	Total glutathione
**Fatty liver**					**β (95% CI)**		
**Estimated liver fat %**							
	M2c	463*	**0.12 (0.04, 0.20)**	-0.04 (-0.13, 0.04)	**0.11 (0.03, 0.19)**	**0.11 (0.03, 0.19)**	0.00 (-0.07, 0.08)
**CODAM**	M2f	435*	**0.12 (0.04, 0.20)**	**-0.09 (-0.17, -0.01)**	**0.08 (0.00, 0.15)**	0.04 (-0.04, 0.12)	-0.01 (-0.10, 0.08)
	M2g	432*	**0.06 (0.02, 0.10)**	-0.01 (-0.05, 0.03)	-0.02 (-0.06, 0.03)	**0.04 (0.00, 0.08)**	**0.07 (0.03, 0.10)**
**Liver fat %**							
	M2c	351	**0.14 (0.01, 0.26)**	0.08 (-0.04, 0.19)	**0.12 (0.01, 0.22)**	0.08 (-0.03, 0.20)	-0.02 (-0.14, 0.10)
**DMS**	M2d	327	**0.13 (0.01, 0.26)**	0.06 (-0.05, 0.17)	0.09 (-0.01, 0.20)	0.04 (-0.08, 0.15)	-0.03 (-0.16, 0.09)
	M2e	327	**0.14 (0.01, 0.27)**	0.09 (-0.03, 0.20)	**0.11 (0.00, 0.22)**	0.08 (-0.03, 0.20)	-0.02 (-0.14, 0.10)
	M2f	349	**0.12 (0.00, 0.24)**	0.10 (-0.01, 0.20)	**0.10 (0.00, 0.20)**	0.04 (-0.07, 0.15)	0.00 (-0.11, 0.12)
					**OR (95% CI)**		
**Fatty liver**							
	M2c	418*	**1.46 (1.17, 1.84)**	0.94 (0.75, 1.18)	0.92 (0.73, 1.14)	1.10 (0.88, 1.38)	1.10 (0.91, 1.37)
**CODAM**	M2f	406*	**1.46 (1.16, 1.87)**	0.92 (0.73, 1.16)	0.88 (0.69, 1.10)	1.08 (0.85, 1.38)	1.11 (0.85, 1.45)
	M2g	391*	**1.48 (1.17, 1.89)**	0.97 (0.77, 1.20)	0.90 (0.71, 1.13)	1.07 (0.85, 1.35)	1.18 (0.97, 1.49)
	M2c	351	1.29 (0.95, 1.79)	1.04 (0.80, 1.34)	1.25 (0.99, 1.59)	**1.32 (0.01, 0.73)**	1.23 (0.94, 1.65)
**DMS**	M2d	327	**1.51 (1.06, 2.18)**	1.22 (0.93, 1.60)	1.23 (0.94, 1.60)	1.19 (0.90, 1.59)	0.78 (0.42, 1.15)
	M2e	327	**1.45 (1.04, 2.07)**	1.28 (0.98, 1.66)	1.26 (0.99, 1.63)	**1.32 (1.00, 1.74)**	0.79 (0.43, 1.17)
	M2f	349	**1.55 (1.07, 2.27)**	**1.34 (1.00, 1.79)**	1.25 (0.97, 1.63)	1.27 (0.94, 1.73)	0.87 (0.47, 1.29)

Standardized beta coefficients (95% confidence intervals) are shown. All models are adjusted for age, sex, glucose metabolism status, smoking status, alcohol and coffee consumption, physical activity, progenitor SAA, protein intake and total energy consumption (in models with plasma methionine or total cysteine as main exposure), height (in models with DXA-derived measures as main outcome) and, were applicable, for the lag-time before MRI and DXA scans. M2c: M2 with additional adjustment for plasma or serum creatinine. M2d: M2 with additional adjustment for lean mass. M2e: M2 in sample with no missing data on lean mass. M2f: M2 with additional adjustment for plasma glucose and lipid, prevalent CVD, medication, systolic blood pressure and kidney function. M2g: M2 with additional adjustment for insulin resistance. *: in models with cystathionine, one participant was excluded because of an extreme value. Figures in bold represent statistically significant associations (p<0.05).

Table S5: Associations between each SAA and measures of obesity and specific fat depots with additional adjustment for branched-chain amino acids and tyrosine (sensitivity analyses)

	Model	Sample size	Methionine	Total homocysteine	Cystathionine	Total cysteine	Total glutathione
**General obesity**					**β (95% CI)**		
**BMI**							
	M2h	372	0.00 (-0.12, 0.12)	**0.12 (0.03, 0.23)**	0.05 (-0.05, 0.15)	**0.25 (0.15, 0.35)**	-0.05 (-0.16, 0.06)
**DMS**	M2i	372	-0.04 (-0.18, 0.09)	**0.14 (0.04, 0.24)**	0.07 (-0.02, 0.17)	**0.24 (0.14, 0.34)**	-0.04 (-0.14, 0.07)
	M2j	372	-0.08 (-0.23, 0.06)	**0.13 (0.03, 0.23)**	0.05 (-0.05, 0.15)	**0.25 (0.15, 0.35)**	-0.04 (-0.15, 0.06)
**Fat percentage**							
	M2h	368	-0.01 (-0.10, 0.08)	0.07 (-0.00, 0.14)	0.03 (-0.05, 0.10)	**0.17 (0.10, 0.25)**	-0.06 (-0.14, 0.02)
**DMS**	M2i	368	-0.09 (-0.19, 0.01)	**0.08 (0.00, 0.15)**	0.03 (-0.04, 0.10)	**0.17 (0.09, 0.24)**	-0.05 (-0.13, 0.03)
	M2j	368	-0.10 (-0.21, 0.01)	**0.07 (0.00, 0.15)**	0.03 (-0.05, 0.10)	**0.17 (0.10, 0.25)**	-0.05 (-0.13, 0.03)
**Total fat mass**							
	M2h	347	0.07 (-0.05, 0.19)	**0.14 (0.05, 0.24)**	0.07 (-0.03, 0.17)	**0.24 (0.14, 0.33)**	-0.06 (-0.16, 0.05)
**DMS**	M2i	347	-0.07 (-0.20, 0.06)	**0.14 (0.05, 0.24)**	0.06 (-0.04, 0.15)	**0.23 (0.13, 0.33)**	-0.05 (-0.15, 0.05)
	M2j	347	-0.04 (-0.19, 0.10)	**0.15 (0.05, 0.24)**	0.07 (-0.03, 0.16)	**0.23 (0.14, 0.33)**	-0.05 (-0.15, 0.05)
**Peripheral adipose tissue**							
**Biceps and triceps skinfolds**							
	M2h	368	-0.05 (-0.15, 0.06)	**0.13 (0.04, 0.21)**	0.01 (-0.07, 0.10)	**0.21 (0.12, 0.30)**	-0.09 (-0.19, 0.01)
**DMS**	M2i	368	-0.09 (-0.21, 0.03)	**0.13 (0.04, 0.22)**	0.02 (-0.06, 0.11)	**0.20 (0.11, 0.29)**	-0.08 (-0.18, 0.01)
	M2j	368	-0.11 (-0.24, 0.01)	**0.13 (0.05, 0.22)**	0.01 (-0.07, 0.10)	**0.21 (0.12, 0.30)**	-0.09 (-0.18, 0.01)
**Limb fat mass**							
	M2h	347	0.11 (-0.01, 0.22)	**0.16 (0.07, 0.25)**	0.07 (-0.02, 0.16)	**0.22 (0.12, 0.31)**	-0.06 (-0.16, 0.03)
**DMS**	M2i	347	-0.01 (-0.13, 0.12)	**0.15 (0.06, 0.24)**	0.05 (-0.04, 0.14)	**0.22 (0.12, 0.31)**	-0.07 (-0.16, 0.03)
	M2j	347	0.05 (-0.09, 0.18)	**0.16 (0.07, 0.25)**	0.07 (-0.02, 0.16)	**0.22 (0.12, 0.31)**	-0.06 (-0.16, 0.04)

Table S5 (continued)

	Model	Sample size	Methionine	Total homocysteine	Cystathionine	Total cysteine	Total glutathione
**Subcutaneous adipose tissue**					**β (95% CI)**		
**SAT**							
**DMS (MRI)**	M2h	370	0.10 (-0.02, 0.23)	0.06 (-0.04, 0.17)	0.06 (-0.04, 0.16)	**0.18 (0.07, 0.28)**	0.01 (-0.10, 0.13)
	M2i	370	-0.04 (-0.18, 0.10)	0.06 (-0.04, 0.16)	0.04 (-0.05, 0.14)	**0.17 (0.07, 0.27)**	0.01 (-0.10, 0.13)
	M2j	370	-0.01 (-0.16, 0.14)	0.07 (-0.03, 0.17)	0.06 (-0.04, 0.16)	**0.18 (0.07, 0.28)**	0.02 (-0.09, 0.13)
**Central adiposity**							
**Waist**							
	M2h	372	-0.02 (-0.13, 0.09)	0.09 (-0.01, 0.18)	0.05 (-0.05, 0.14)	**0.17 (0.08, 0.26)**	-0.03 (-0.13, 0.07)
**DMS**	M2i	372	-0.08 (-0.20, 0.05)	**0.09 (0.00, 0.18)**	0.07 (-0.02, 0.16)	**0.17 (0.08, 0.26)**	-0.02 (-0.12, 0.08)
	M2j	372	-0.12 (-0.25, 0.02)	0.09 (-0.00, 0.18)	0.05 (-0.04, 0.14)	**0.17 (0.08, 0.26)**	-0.02 (-0.12, 0.08)
**Suprailiacal and subscapular skinfolds**							
	M2h	368	-0.01 (-0.13, 0.11)	0.05 (-0.05, 0.15)	0.04 (-0.06, 0.14)	**0.16 (0.06, 0.26)**	-0.06 (-0.17, 0.05)
**DMS**	M2i	368	-0.08 (-0.21, 0.06)	0.06 (-0.04, 0.16)	0.04 (-0.05, 0.14)	**0.15 (0.05, 0.26)**	-0.05 (-0.16, 0.06)
	M2j	368	-0.11 (-0.25, 0.04)	0.06 (-0.04, 0.15)	0.02 (-0.08, 0.12)	**0.16 (0.06, 0.26)**	-0.05 (-0.16, 0.06)
**VAT**							
	M2h	369	-0.05 (-0.15, 0.05)	0.08 (-0.01, 0.16)	0.02 (-0.06, 0.11)	**0.11 (0.03, 0.20)**	-0.02 (-0.11, 0.07)
**DMS (MRI)**	M2i	369	-0.10 (-0.22, 0.02)	**0.09 (0.01, 0.17)**	0.05 (-0.03, 0.13)	**0.11 (0.02, 0.19)**	-0.01 (-0.10, 0.08)
	M2j	369	**-0.16 (-0.28, -0.04)**	0.08 (-0.00, 0.16)	0.02 (-0.06, 0.11)	**0.11 (0.03, 0.20)**	-0.02 (-0.11, 0.08)

Table S5 (continued)

**Fatty liver**							
**Liver fat %**							
	M2h	351	-0.02 (-0.16, 0.11)	0.06 (-0.05, 0.16)	0.06 (-0.04, 0.16)	0.07 (-0.04, 0.18)	-0.04 (-0.16, 0.07)
**DMS**	M2i	351	-0.07 (-0.23, 0.10)	0.08 (-0.03, 0.18)	0.10 (-0.01, 0.20)	0.07 (-0.04, 0.18)	-0.02 (-0.14, 0.10)
	M2j	351	-0.16 (-0.32, 0.00)	0.06 (-0.05, 0.16)	0.06 (-0.04, 0.16)	0.07 (-0.04, 0.18)	-0.04 (-0.16, 0.07)
					**OR (95% CI)**		
**Fatty liver**							
	M2h	351	1.23 (0.85, 1.78)	1.20 (0.92, 1.56)	1.19 (0.92, 1.54)	1.29 (0.97, 1.71)	0.79 (0.44, 1.13)
**DMS**	M2i	351	1.05 (0.68, 1.61)	1.24 (0.96, 1.60)	1.27 (0.99, 1.63)	1.28 (0.97, 1.68)	0.80 (0.43, 1.19)
	M2j	351	0.98 (0.62, 1.52)	1.20 (0.92, 1.56)	1.19 (0.92, 1.55)	1.27 (0.96, 1.69)	0.79 (0.42, 1.14)

Standardized beta coefficients (95% confidence intervals) are shown. Adjusted for age, sex, glucose metabolism status, smoking status, alcohol and coffee consumption, physical activity, progenitor SAA, protein intake and total energy consumption (in models with plasma methionine or total cysteine as main exposure), height (in models with DXA-derived measures as main outcome) and, were applicable, for the lag-time before MRI and DXA scans. M2h: M2 with additional adjustment for plasma concentrations of leucine, isoleucine and valine. M2i: M2 with additional adjustment for plasma concentrations of tyrosine. M2j: M2 with additional adjustment for plasma concentrations of leucine, isoleucine, valine and tyrosine. Figures in bold represent statistically significant associations (p<0.05).

Table S6: Analyses stratified by sex (sensitivity analyses)

	Model	P-value interaction	Sex	Sample size	Total cysteine
					**β (95% CI)**
**General obesity**					
**BMI**					
**CODAM**	M2k	0.10	Males	287	**0.14 (0.03, 0.26)**
			Females	182	**0.26 (0.10, 0.43)**
**DMS**	M2k	0.24	Males	193	**0.20 (0.07, 0.33)**
			Females	158	**0.27 (0.12, 0.43)**
**Central adiposity**					
**Suprailiacal and subscapular skinfolds**					
**CODAM**	M2k	**0.04**	Males	284	0.09 (-0.03, 0.21)
			Females	179	**0.31 (0.15, 0.47)**
**DMS**	M2k	0.88	Males	197	**0.18 (0.04, 0.32)**
			Females	171	0.14 (-0.01, 0.30)

Standardized beta coefficients (95% confidence intervals) are shown. Adjusted for age, glucose metabolism status, smoking status, alcohol and coffee consumption, physical activity, plasma cystathionine, protein intake and total energy consumption. * In models with cystathionine, one participant was excluded because of an extreme value. Figures in bold represent statistically significant associations (p<0.05).
